# Proteins with cognition-associated structural changes in a rat model of aging exhibit reduced refolding capacity

**DOI:** 10.1126/sciadv.adt3778

**Published:** 2025-07-11

**Authors:** Haley E. Tarbox, Audrey Branch, Stephen D. Fried

**Affiliations:** ^1^ Department of Chemistry, Johns Hopkins University, Baltimore, MD 21218, USA.; ^2^ Department of Psychological and Brain Sciences, Johns Hopkins University, Baltimore, MD 21218, USA.; ^3^ T. C. Jenkins Department of Biophysics, Johns Hopkins University, Baltimore, MD 21218, USA.

## Abstract

Cognitive decline during aging represents a major societal burden, causing both personal and economic hardship in an increasingly aging population. Many studies have found that the proteostasis network, which functions to keep proteins properly folded, is impaired with age, suggesting that there may be many proteins that incur structural alterations with age. Here, we used limited proteolysis mass spectrometry, a structural proteomic method, to globally interrogate protein conformational changes in a rat model of cognitive aging. Specifically, we compared soluble hippocampal proteins from aged rats with preserved cognition to those from aged rats with impaired cognition. We identified a couple hundred proteins as having undergone cognition-associated structural changes (CASCs). We report that CASC proteins are substantially more likely to be nonrefoldable than non-CASC proteins, meaning that they typically cannot spontaneously refold to their native conformations after being chemically denatured. These findings suggest that noncovalent, conformational alterations may be general features in cognitive decline.

## Teaser

Up to 7% of rat hippocampal proteins undergo structural changes that associate with age-related decline in spatial learning.

## INTRODUCTION

Cognitive decline in several memory domains is a common feature of aging, even in the absence of neurodegenerative disease. However, cognitive decline is not inevitable; some individuals retain cognitive abilities on par with young adults well into their later decades of life. Identifying molecular features that are retained by these cognitively resilient individuals or those that are altered in impaired subjects can provide opportunities for prevention or treatment of age-related cognitive decline. Individual variability in cognitive aging is not unique to humans. The use of animal models, such as outbred rats, has established that cognitive integrity at older ages is due to retaining functional circuit computations within an aging brain context. For example, in the well-studied medial temporal lobe (MTL) episodic memory circuit, hippocampal subfields do not exhibit overt cell loss in aged subjects with MTL-related memory impairments (*1*), and neuronal connections between hippocampal subfields are generally preserved (*2*, *3*). However, research has provided ample evidence for functional alterations in the hippocampus that are closely tied to age-related cognitive decline (*4*–*6*), which are linked to cellular and molecular changes (*7*–*9*).

In the context of a largely structurally preserved MTL, age-related changes in the proteome emerge as a potential mediator of cognitive circuit dysfunction leading to memory impairment. While protein aggregation plays a causal role in several late-life neurodegenerative diseases that impair cognition, this is largely related to the neurotoxic effects of aggregates. Amyloids have distinctive and stable structures, which make them visible through many methods on several length scales (x-ray crystallography to histological staining). It is notable that relatively few proteins have been shown to misfold and/or aggregate in this marked way. However, proteins can also misfold in an age-dependent or cognition-relevant manner but do so in a way that retains them in a soluble misfolded (*10*) and/or oligomeric (*11*, *12*) form, which would be challenging to detect with most techniques.

The proteostasis network is critical for maintaining proper protein structure in vivo and comprises the processes and factors responsible for maintaining the proper balance of protein synthesis, folding, maintenance, and degradation (*13*). Loss of correctly functioning cellular proteostasis has been shown to arise with age (*14*–*16*), and a combination of chaperone dysfunction, turnover impairment, and posttranslational modification (PTM) accumulation that occurs with age creates the ingredients for proteome-wide protein structural changes, which could cause changes in function. There is already evidence for specific proteins adopting soluble misfolded forms during aging. For example, altered conformations of phosphoglycerate kinase and enolase were found in aged rat brains (*17*) and aged nematodes (*18*), respectively, among other examples (*19*). However, these studies have been limited to single proteins, and age-related protein structural changes have yet to be studied on a proteome scale.

**Fig. 1.**
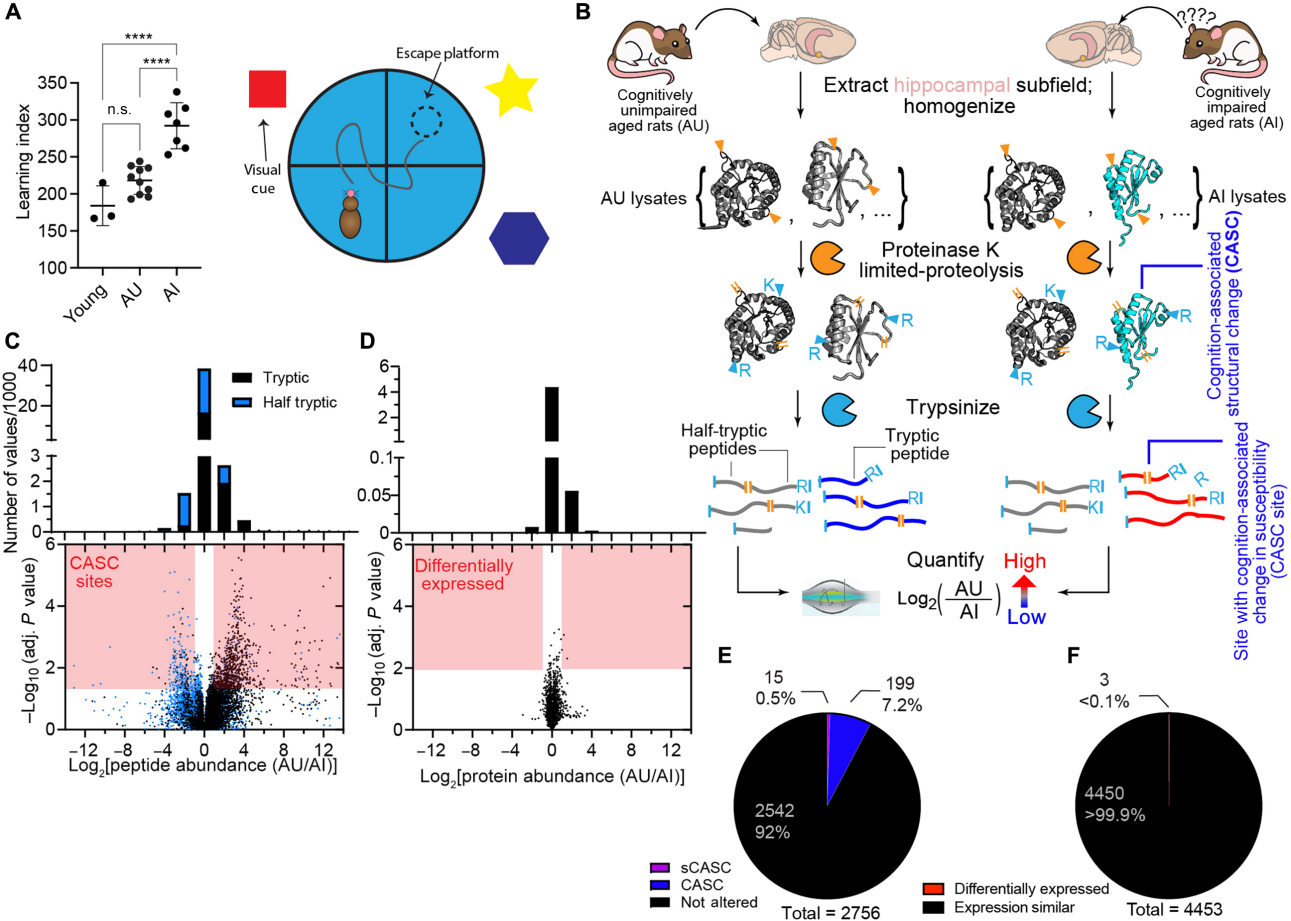
Measuring protein conformational changes associated with age-associated changes in cognition with LiP-MS. (**A**) Learning indexes (*31*) as assessed with the MWM test (shown schematically; right) on the 20 Long-Evans rats in this study. Aged (24 months) unimpaired (AU) rats have learning indexes comparable to young (~6 months) rats, while aged impaired (AI) rats have a significantly higher learning index than both [****P < 0.0001 by analysis of variance (ANOVA) with Brown-Forsythe correction and Tukey’s multiple comparisons test]. n.s., not significant. (**B**) Experimental scheme for LiP-MS. Proteins in hippocampal subfields from AU and AI rats are assessed for structural differences by performing pulse proteolysis with proteinase K (PK) under native conditions, followed by complete digest with trypsin under denaturing conditions. The proteolysis pattern is measured with liquid chromatography MS (LC-MS) and label-free quantification (LFQ). (**C** and **D**) Volcano plots comparing peptide abundances of tryptic (black) and half-tryptic (blue) peptides (C) and protein abundances (D) in the CA1 subfield between AU and AI rats. Dots within red regions are deemed significant on the basis of effect size (>2-fold) and adjusted *P* < 0.05 by *t* test with Welch’s correction for unequal population variance and false discovery rate (FDR) corrected by the Benjamini-Hochberg procedure (C) or based on effect size (>2-fold) and *P* < 0.01 by *t* test with Welch’s correction for unequal population variance (D). Histograms show the number of peptides having abundance ratios in the various ranges. (**E**) Number of proteins with cognition-associated structural changes (CASCs) in the CA1 subfield. CASC proteins have two or more peptides with significant changes. sCASC denotes CASCs from the stricter analysis, which overlap with the general analysis (with one exception). All proteins shown have two or more peptides detected. (**F**) Number of proteins with different measured abundance in AU versus AI subjects in the CA1 subfield.

Limited proteolysis mass spectrometry (LiP-MS) is a structural proteomic technique that is able to distinguish subtle structural changes across many proteins in a complex mixture (Fig. 1B) (*20*, *21*). These structural changes are detected by comparing differences in proteolysis patterns and can arise from conformational changes, differences in ligand binding, PTMs, protein-protein interactions, or oligomeric state. LiP-MS has already been applied in an aging context to study protein structural changes in cerebrospinal fluid from young and aged mice (*22*) and in young and aged yeast extracts (*23*).

In this study, we use LiP-MS to interrogate the structural neuroproteome of the hippocampus in an aging rat model. In this model, genetically variable, outbred aged rats are categorized as being aged and cognitively unimpaired (AU) or aged and cognitively impaired (AI) based on performance in the Morris water maze (MWM) test (Fig. 1A) (*24*). AI rats exhibit subfield-specific alterations in MTL physiology and function, which mirror those observed in aged humans with memory impairments and have successfully guided human clinical studies (*25*, *26*). AU rats execute MTL-dependent tasks on par with young animals, while exhibiting age-adaptive molecular (*7*, *27*), cellular (*28*, *29*), and circuit mechanisms (*2*, *4*, *30*). Thus, cognitively characterized aged Long-Evans rats can serve as a powerful model system for assessing how individual differences in protein structures may contribute to or prevent age-related cognitive decline. We reasoned that the subfields of the hippocampus in this model could be particularly relevant contexts for assessing changes in protein folding, given the extensive characterization of this circuit in both young and aged subjects. Moreover, we focus on a comparison between two aged populations in this study to isolate molecular factors associated with a cognitive phenotype, in contrast to comparing young and old populations that could convolve other factors.

In this study, we assess protein structural changes in the rat hippocampus, covering 2756 distinct CA1 hippocampal proteins, and we report evidence that many (214) proteins can have soluble structural changes that differ between cognitive phenotypes. We identified proteins with cognition-associated structural changes (CASCs) by comparing the proteolysis patterns between these cohorts. A number of these proteins have been implicated in memory, aging, and synaptic health, whereas many others represent previously unexplored targets for further study or therapeutics. Perhaps most notably, we find that CASC proteins are generally nonrefoldable; that is, they often are unable to spontaneously return to their native conformations following global denaturation. Nonrefoldability explains more of the CASCs than oxidation or phosphorylation levels, suggesting that noncovalent, conformational alterations may be a general feature underlying cognitive decline. Because these structural changes are observed in a background wherein all animals are of the same age, this study reports directly on proteins that may be relevant to cognition. More broadly, our findings suggest that many proteins may misfold in more subtle ways and, unlike amyloids, remain soluble yet still be relevant to deterioration in cognitive abilities.

## RESULTS

### CASCs are pervasive in the rat hippocampal proteome

Long-Evans rats were aged to 24 months in a colony with a controlled diet and environment. Young animals in this population (6 months) exhibit proficient spatial memory as evidenced by their performance on the MWM test, quantified by the learning index scale described previously (*31*). Aged rats, on the other hand, exhibit a wider spectrum in their capacity to perform these tasks effectively (Fig. 1A) with some individuals exhibiting impaired spatial learning abilities relative to young subjects. These AI rats have been shown to have MTL circuit dysfunction including subfield-specific alterations in synaptic density (*2*), disruption of excitatory/inhibitory balance (*4*, *25*), and baseline and learning-related transcription (*7*). Our study included 7 AI rats, 10 AU rats, and 3 young rats. Approximately 2 weeks after MWM cognitive assessment, hippocampal tissue was extracted and dissected into dentate gyrus (DG), CA3, and CA1 subfields. We focused on the hippocampus because it is closely associated with spatial learning and memory in rats (*32*, *33*).

The subfields were Dounce homogenized into a native buffer without detergents, cellular debris and membranes were depleted by centrifugation, and the soluble fractions were subjected to LiP with proteinase K (PK; 1:100, w/w) for 1 min (Fig. 1B). This treatment enables cleavage to occur only at solvent-accessible locations within proteins and thereby encodes structural information about the conformational ensemble of each protein into cut sites. To determine the locations of these cut sites, the samples are trypsinized, and the resulting peptides identified through liquid chromatography tandem MS (LC-MS/MS). In particular, sequenced peptides for which one of the termini can be assigned to a PK cleavage (orange lines in Fig. 1B, called half-tryptic peptides) because it does not follow trypsin’s cleavage rules specify a site of solvent accessibility in the parental protein structure. Changes in abundance of the resulting half-tryptic (and tryptic) peptides can be interpreted in terms of persistent structural changes at the corresponding sites that remained following brain tissue extraction and homogenization. Here, we assess structural changes in hippocampal proteins between young, AU, and AI cohorts by performing label-free quantification (LFQ) on the 20 (or 19) samples, and studies of this kind were performed on each of these three hippocampal subfields separately. In general, slightly fewer peptides/proteins are identified in LiP runs (fig. S1, A to F), since the treatment degrades some protein regions, but overall similar numbers of peptides/proteins were identified across runs, subfields, and cohorts. We also performed a parallel “trypsin-only” set of samples in which the LiP step was withheld; the resulting quantifications provide a means to assess protein abundance differences between AU and AI rats and to normalize the peptide abundance ratios from the LiP samples with overall protein-level abundance ratios. A relatively small fraction of the identified peptides in the trypsin-only control samples were of the half-tryptic variety (8.2 ± 0.8%), in contrast to that of LiP samples (46.7 ± 6.5%; fig. S1, G to I). This suggests that most of the half-tryptic peptides we sequenced correspond specifically to cleavage from structural probing by PK (and not by sample degradation or endogenous protein decay), which was possible due to an amended LiP-MS protocol in which extracts were treated with a specific cocktail of protease inhibitors to mitigate degradation in the lysate without interfering with PK (see Materials and Methods).

In Fig. 1C, we show a normalized peptide-level volcano plot summarizing the results from this experiment in the CA1 subfield; on these diagrams, each point represents a confidently identified and quantified peptide. We quantified 43,482 peptides, of which 4894 (11.3%) were above our thresholds [twofold effect size, adjusted *P* < 0.05 by two-tailed *t* test with Welch’s correction with per-protein multiple hypothesis correction using the Benjamini-Hochberg method as implemented in a modified version of the tool FragPipe LiP Processor (FLiPPR) (*34*)] to be considered significantly enriched in either AU or AI hippocampi. Next, we assigned these peptides to their parental proteins, and if we detected two or more uniquely mappable peptides with significantly altered proteolytic susceptibility, then the protein is labeled a CASC protein. We note that because learning index is numerical (Fig. 1A), it is also possible to test the relationship between a peptide’s abundance and cognition levels through regression rather than by *t* test. Initial tests revealed, however, that this approach generally provided lower statistical power compared to one using the AU/AI categorization (fig. S2).

Analysis of these data is complicated for three reasons: First, since each replicate is from a separate, outbred organism, this dataset has higher variability than might be expected from a genetically homogenous sample (fig. S3); second, because LiP-MS is fundamentally a peptide-centric form of proteomics, it is not afforded the statistical power derived from averaging together all peptides for a protein; and third, LiP-MS can produce data with many missing values especially when major structural changes occur (*35*). To minimize false positives brought about by this variability, we not only implemented a false discovery correction procedure (*34*) but also explored several ways to impute missing values and filter features with missing data. Ultimately, we analyzed the data two ways (see details in Materials and Methods). First, we use a “strict” analysis that does not impute any values for missing data and typically filters out the features with more missing values. This procedure identified 15 strict CASCs (sCASCs) (table S1). This strict approach reduces false discovery effectively, since in three separate null analyses in which the AU/AI labels were randomly permuted across the replicates, only one protein was labeled CASC [table S2; false discovery rate (FDR) estimated to be ≤6%]. In LiP experiments, missing data can arise from real protein structural differences, but they can also be due to acquisition or other factors. This balance is why we chose to also implement a more “general” analysis, in which imputation of missing values is performed when certain conditions are met (see Materials and Methods). We varied the filters to either discard a feature or impute its missing values to optimize our sensitivity to detect CASCs subject to the constraint of minimizing false discoveries as estimated by performing three null analyses with randomly permuted labels (table S3). We estimate the FDR of the general analysis is 20% in CA1 (table S2), which is high but likely the best that is possible for this high-variability, missing data–rich dataset. This means that while any individual CASC may need additional experimental validation, we are not missing the majority of CASCs and can still draw conclusions about the types of proteins that are likely to be CASC. On the other hand, the 15 sCASCs can be considered valid as is. In addition, it is also possible that structural changes occurring in domains not accessible to LiP in either conformation will not be detected by this method, so there is a chance of false negatives.

In the general analysis, 214 proteins are CASC in CA1 (Fig. 1E), representing 7.8% of the total proteins assessed. Of the 214 CASCs, 14 are sCASCs, meaning that all but one sCASC are also identified in the general analysis. We moreover verified that LiP-MS quantifications are highly reproducible on hippocampal extracts in a separate study (fig. S4) in which we compared differences in measured peptide levels across different injections (measuring instrumental variability), different LiP sample preparations (measuring variability from the LiP protocol), and different subjects (measuring biological variability).

We detected markedly fewer differences in the CA3 subfield between the AU and AI cohorts (fig. S1, J to M), and considering the null analyses (table S2), we do not find that our study has sufficient statistical power to confidently identify CASCs in the CA3 subfield. While one explanation of these results could be that the CA3 preparations had higher within-sample variation, we found instead that the within-sample variation for CA3 is comparable to the variation observed for the CA1 preparations (fig. S3, D to F and J to L). This suggests that the relative lack of CASC proteins in CA3 is unlikely to be a result of technical or LiP-related reproducibility issues and rather that proteostasis may be more robust in CA3. These observations are qualitatively consistent with prior work suggesting that the CA3 subfield of the hippocampus may have some relative protection from stresses related to aging, such as oxidative stress, compared to the CA1 subfield (*36*, *37*). We see more differences within the DG region (fig. S1, N to Q); however, our samples in this subfield showed higher within-group variability (fig. S3, G to I) compared to the CA1 and CA3 regions, and because of this, we do not consider the data of sufficient quality to draw confident conclusions about which proteins are CASCs in the DG subfield.

It is notable to point out that at the protein-level, there are very few abundance differences between the AU and AI cohorts (Fig. 1D). In CA1, only three proteins are differently expressed (Fig. 1F). Hence, structural proteomic methods can detect differences on more proteins than a “standard” omics approach, which only reports on protein abundances, when comparing two wild-type populations that vary in their cognitive phenotype.

### CASCs are less capable of spontaneously refolding after denaturation

**Fig. 2.**
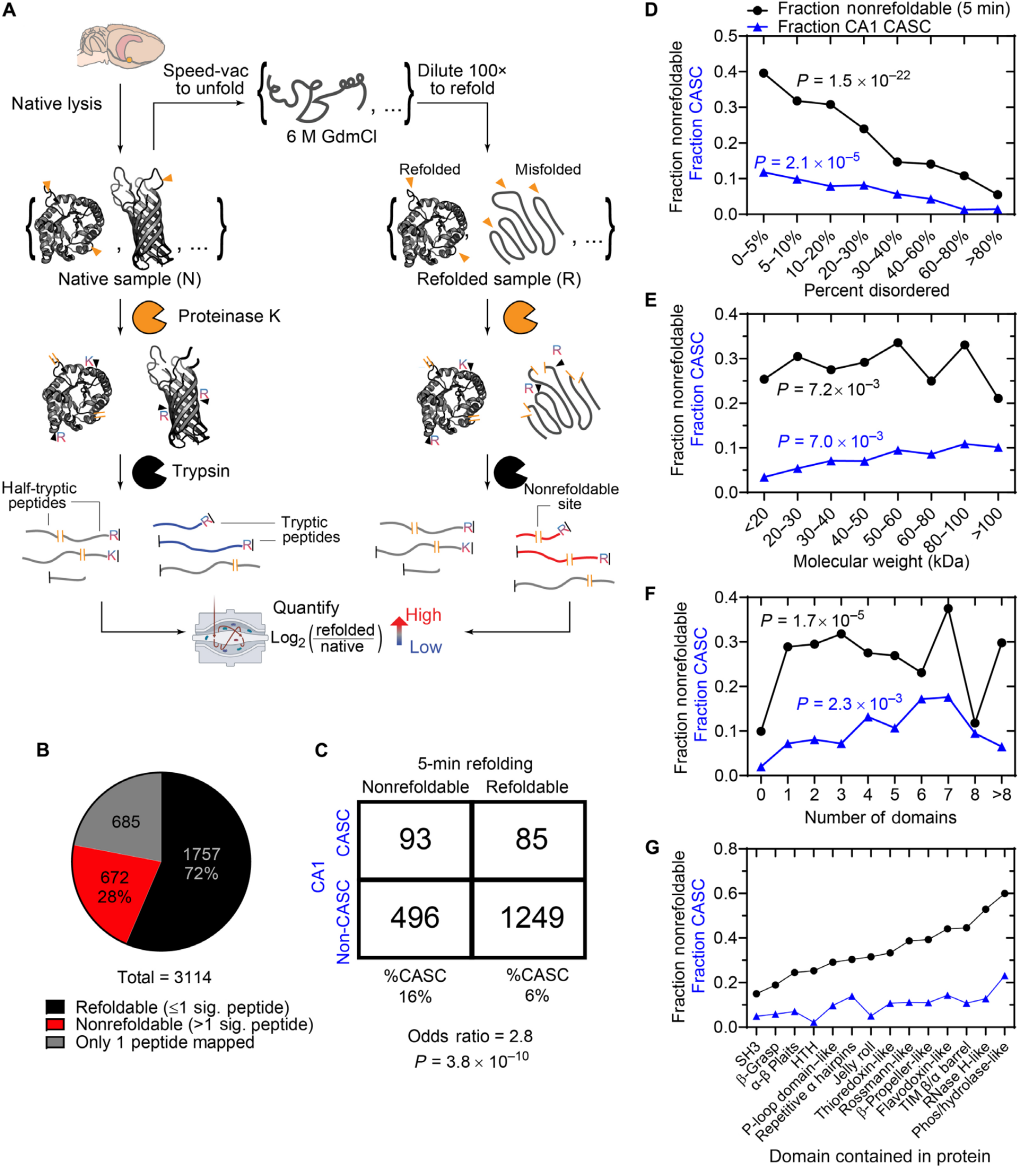
Similarities between nonrefoldable proteins and CASC proteins. (**A**) Experimental scheme for LiP-MS to assess refoldability. Proteins in the young rat hippocampus are unfolded by incubation in 6 M GdmCl and are refolded by 100-fold dilution into a native buffer. The structures of the native proteins and their refolded forms are compared by performing pulse proteolysis with PK, followed by complete digestion with trypsin, and the proteolysis profiles are measured. (**B**) Number of refoldable (black) and nonrefoldable (red) proteins in the hippocampus. Nonrefoldable proteins have two or more peptides with significant changes following refolding. Proteins with only one peptide mapped are discounted (gray). (**C**) Contingency table comparing refoldability status in the global refolding studies and CASC status in the cognition studies. Refolding after 5 min and CASC status in the CA1 were used as the reference conditions, respectively. Shown below the table are the marginal frequencies of CASC status among (non)refoldable proteins, the odds ratio, and the *P* value against the null hypothesis of independence, according to Fisher’s exact test. (**D** to **G**) Plots show the fraction of proteins that are nonrefoldable (black; 5 min) or CASC (blue; in CA1) as a function of (D) their percent disorder according to Metapredict (*40*), (E) their molecular weight, and (F) their number of domains. (G) assesses proteins based on whether they contain a domain of a given topology [based on Evolutionary Classifications of Domains (ECOD) (*94*)] although these categories are not mutually exclusive since some proteins contain multiple domains. *P* values (according to chi-square test) against the null hypothesis that nonrefoldability/CASC status is independent of the categorical variable in question are provided.

Previous work of ours (*35*, *38*) has applied LiP-MS to address what might outwardly appear to be an unrelated question: Which proteins are incapable of spontaneously refolding to their native structures following complete denaturation? In these experiments, an extract is subjected to a global unfolding-refolding cycle (Fig. 2A), in which proteins are first incubated overnight in 6 M guanidinium chloride (GdmCl) and 10 mM dithiothreitol (DTT) and then diluted 100-fold to initiate refolding. Following several refolding times, the proteome-wide refolding reactions are probed by LiP and the resulting proteolysis pattern compared to that of native (unperturbed) samples that were not subjected to an unfolding-refolding cycle. We consider these experiments a probe of a protein’s spontaneous (unassisted) refolding capacity because chaperone and adenosine 5′-triphosphate (ATP) concentrations in the diluted lysates are too low to play a substantial role unless they are supplemented to cellular-like levels (*39*). Our previous work demonstrated that after 5 min (a time that allows many proteins sufficient time to refold but before considerable degradation), 60% of *Escherichia coli* proteins (*35*) and 76% of yeast proteins can refold (*38*). We performed similar global refolding assays on hippocampal extracts from young rats and found an overall refolding rate after 5 min (72%) more similar to that of yeast (Fig. 2B); hence, we continued to use this time point as the reference condition (fig. S5C). As previously, to be labeled nonrefoldable, a protein needed to have at least two sites with altered susceptibility (twofold effect size, adjusted *P* < 0.05 by two-tailed *t* test with Welch’s correction and FDR correction using the Benjamini-Hochberg method) in the refolded form. At dilute concentrations of 0.1 mg/ml, global refolding reactions on the hippocampal extract could proceed without any detectable level of precipitation (fig. S5A), and the refolded proteins were, on the whole, more susceptible to LiP (fig. S5B), suggesting the formation of soluble misfolded conformations. The abundance of LiP peptides in refolding reactions from biological replicates showed modest variability (fig. S3, A to C); the 5-min refolding reactions have a median coefficient of variation of 19% (fig. S3B); and other quality controls were acceptable, such as consistent peptide/protein IDs across replicates, low incidence (10.9 ± 0.2%) of half-tryptic peptides in trypsin-only controls, and an expected frequency (49.2 ± 4.0%) of half-tryptic peptides in LiP samples (fig. S5, D to F). It is notable that the variation across refolding reactions is much lower than the biological variation we encountered in the aging study (range of medians from refolding study, 0.18 to 0.22; range of medians from aging study, 0.29 to 0.59; fig. S3).

To compare the outcomes of these two experiments, we considered each protein whose refoldability and CASC status (in CA1) could be simultaneously assessed. The results in the contingency table (Fig. 2C) show that these statuses are highly associated (*P* value of 3.8 × 10^−10^ by Fisher’s exact test). For instance, refoldable proteins have only a 6% likelihood of being CASC, but nonrefoldable proteins have a 16% (2.7-fold greater) likelihood of being CASC. Among sCASCs, of the 14 sCASCs (of 15 total) that were detected in the refoldability experiment, 9 are nonrefoldable. This 64% nonrefoldability rate is much higher than the overall 28% nonrefoldability rate seen for the whole proteome.

Some structural parameters, such as disorder content, display similar trends for CASC and nonrefoldable proteins. For example, as the protein’s disorder content [as calculated by Metapredict (*40*)] increases, the likelihood of a protein being classified as CASC or nonrefoldable generally decreases (Fig. 2D). In contrast, for molecular weight, while the nonrefoldable trend is statistically significant against the null hypothesis, there is no general trend. However, as molecular weight increases, we do find a monotonic increase in the likelihood that a protein is a CASC (Fig. 2E). In the rat brain, the exact number of globular domains is not a good predictor for refoldability or CASC status; however, nonrefoldable and CASC proteins are more likely to have at least one globular domain instead of zero (Fig. 2F).

One potential limitation of our metric to ascribe nonrefoldable or CASC status (two or more sites on a protein with altered susceptibility) is that proteins with more peptides mapped to them are more likely to have altered peptides. However, we address this by performing the Benjamini-Hochberg method for multiple hypothesis correction for each protein. Another way this potential bias can be evaluated is by assessing all the peptide sites without first assigning them to proteins (*35*). For instance, we find that sites from proteins with 60 to 100% disorder have the lowest propensity to be structurally altered following unfolding/refolding, implying that the high incidence of nonrefoldability among this class of proteins is not due to coverage bias, as this trend recapitulates what was seen on the protein level (fig. S6A). Similarly, we find that peptides from proteins with a percent disorder between 60 and 100% also have the lowest propensity to be CASC sites (altered susceptibility between AU and AI) (fig. S6B). On the basis of the frequency of significant sites, we would assess that the relationship between molecular weight and CASC status is less robust but the relationship between number of domains and CASC status is more robust (fig. S6, C to F).

An important way that nonrefoldability and CASC statuses cohere with one another is by noting which types of domain topologies are contained in these proteins. Among proteins we observe in the rat hippocampus, all-α and all-β domains [such as helix-turn-helix (HTH), Src homology 3 (SH3) folds, β-grasp (the ubiquitin-like domain)] typically refold efficiently, but larger α/β domains—particularly those involved in metabolic processes, such as triose-phosphate isomerase (TIM) barrels, and flavodoxin-like domains—have low refolding capacity (Fig. 2G). This mimics what we observed in a recent study investigating refoldability in the yeast proteome (*38*). Moreover, the same types of domains that exhibit higher nonrefoldability also tended to have high propensity to become structurally perturbed in a cognition-dependent manner (Fig. 2G, compare blue and black traces). For example, 60% of proteins that contain a phosphorylase/hydrolase-like domain and 44% of proteins that contain a flavodoxin-like domain are nonrefoldable. Over 23 and 14%, respectively, of proteins containing these same domains are CASC proteins. On the other hand, proteins containing domains such as β-grasp or SH3 are overwhelmingly refoldable (<20% nonrefoldable) and are also unlikely to be CASC proteins (<6%). This trend appears robust on the basis of similar nonrefolding/CASC propensities being observed at both the protein and peptide levels (fig. S6, G and H).

### Critical proteins emerge as CASC proteins

**Fig. 3.**
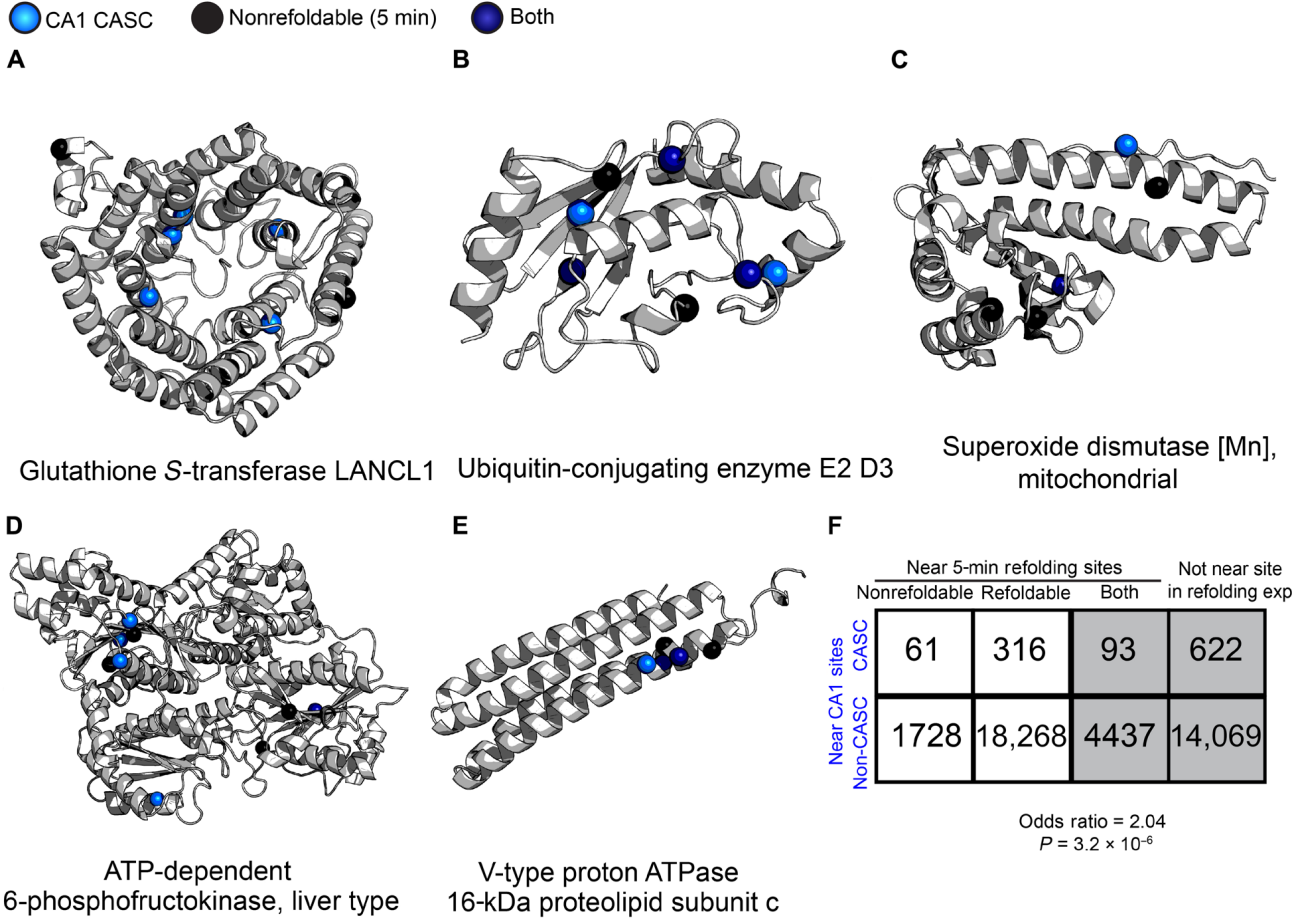
Colocalization between nonrefoldable sites and CASC sites. (**A** to **E**) AlphaFold2 structural models displayed for (A) glutathione *S*-transferase LANCL1 (Q9QX69), (B) ubiquitin-conjugating enzyme E2 D3 (P61078), (C) superoxide dismutase [Mn], mitochondrial (SOD2; P07895) (disordered residues 1 to 15 not displayed), (D) ATP-dependent 6-phosphofructokinase, liver type (Pfkl; P30835), and (E) V-type proton ATPase 16-kDa proteolipid subunit c (Atp6v0c; P63081) showing the locations of residues that are structurally altered (based on changes in proteolytic susceptibility) after 5 min of refolding from denaturant in global refolding assays (black spheres) or structurally altered in CA1 between AU and AI rats (light-blue spheres) in the sCASC dataset (ubiquitin-conjugating enzyme E2 D3 and phosphofructokinase have additional significant sites found in the dataset with imputation, also shown here). Residues that are structurally altered in both experiments are shown as dark-blue spheres. Indicated positions represent cut sites for half-tryptic peptides and midpoint residue for tryptic peptides. (**F**) Contingency table showing the number of sites that are (non)refoldable in global refolding assays and/or structurally altered in a cognition-associated manner. To be included, a site identified in the cognition experiment had to be within five residues on either side of a site identified in the refolding experiment. Gray represents sites excluded from the analysis because they were not identified in the refolding experiment or were near both a nonrefoldable and a refoldable site. *P* = 3.2 × 10^−6^ by Fisher’s exact test.

While looking at bulk trends of the characteristics of proteins is instructive in getting a sense of what types of proteins are likely to have structural differences between the AU and AI populations, we investigated several proteins in greater detail. We modeled several proteins, all of which are sCASCs and nonrefoldable. We modeled sites of nonrefoldability (shown in black), CASC (shown in light blue), or sites that appeared as significant in both experiments (dark blue) onto AlphaFold2 structures, which were used for consistency (*41*). For half-tryptic peptides, the residue associated with the PK cut is shown, whereas for tryptic peptides, the middle residue of the sequenced tryptic fragment is shown. As a note, significant CASC sites are shown from both the sCASC and general CASC datasets, but all modeled sCASC sites are also general CASC sites with one exception. For example, glutathione *S*-transferase LANCL1 (UniProt accession Q9QX69) is an sCASC that is known to be important in the defense of neurons against oxidative stress. Its depletion has been shown to increase reactive oxygen species and mitochondrial dysfunction (*42*). In the case of LANCL1, the sites that are different following complete unfolding and refolding are not colocalized with the sites that differ between AU and AI samples (Fig. 3A).

We also modeled the ubiquitin-conjugating enzyme E2 D3 (P61078) (Fig. 3B). In this case, although the protein is relatively small, the CASC and nonrefoldability sites appear to be near to each other in space, and two significant sites are at the same exact location. This enzyme is part of the ubiquitin-proteasome system (UPS), which is one of the main ways proteins are degraded in eukaryotic cells. Specifically, E2 enzymes conjugate ubiquitin and transfer it to a substrate (brought by E3 enzymes) to tag it for degradation by the proteasome (*43*).

We modeled three additional proteins, which have some connections to aging and/or cognition. Superoxide dismutase [Mn], mitochondrial (SOD2; P07895; Fig. 3C), is found in the mitochondria and converts superoxide, a reactive oxygen species, to the less-dangerous hydrogen peroxide. Mutations in SOD2 can lead to amyotrophic lateral sclerosis (ALS), a neurodegenerative disease (*44*). SOD2 knockouts in astrocytes have also been shown to cause decreases in long-term potentiation and spatial working memory in male mice (*45*). In addition, a cross of an Alzheimer’s disease mouse model with a SOD2-overexpressing model resulted in lower superoxide levels and prevented learning and memory issues in the mice (*46*). Our study reports only on structural differences and not activity; however, it stands to reason that some of these structural changes could lead to differences in activity, and a well-functioning SOD2 is important for neuroprotection. As a note, this protein is the only CA1 sCASC that was not identified as a CASC in the general analysis due to loss of significance in one peptide.

ATP-dependent 6-phosphofructokinase, liver type (Pfkl; P30835; Fig. 3D), is a metabolic enzyme that is part of glycolysis. Decreased glycolytic flux is a common occurrence that is observed in multiple neurodegenerative diseases such as Alzheimer’s disease, Parkinson’s disease, and ALS (*47*). Pfkl is another example where CASC sites and nonrefoldability sites tend to be located near each other spatially. Furthermore, all the CASC and nonrefoldable sites on the V-type proton adenosine triphosphatase (ATPase) 16-kDa proteolipid subunit c (Atp6v0c; P63081; Fig. 3E) are spatially colocalized. Atp6v0c is a subunit of the vacuolar(H^+^)-ATPase, for which one function is creating the proton gradient needed to load synaptic vesicles with cargo (such as neurotransmitters) (*48*). A knockdown of Atp6v0c has been shown to decrease autophagy, an important process for recycling cellular components that are damaged. In addition, autophagy dysfunction is a potential mechanism of neurodegenerative disease (*49*). Last, an E3 ligase, which is important for substrate recognition for proteins to be degraded by the proteasome, that is specific for Atp6v0c is up-regulated in the Alzheimer’s brain, suggesting that increased degradation of Atp6v0c is a potential consequence of (or driver of) neurodegenerative disease (*50*). While this is just a sampling of the CASC proteins that we find, we see that many CASCs have links to cognition or neurodegenerative disease and that their different structures between cognitive phenotypes may be a cause or result of the pressures conferred by aging.

These models demonstrate that nonrefoldable sites and CASC sites are sometimes colocalized, in particular for ubiquitin-conjugating enzyme E2 D3 and Atp6v0c (Figs. 3B, E). On a more global scale, we performed a proximity analysis to determine whether general CA1 CASC sites (from the aging/cognition study) and nonrefoldable sites (from the refolding study) were near one another (within five residues on either side). CASC sites tend to be found uniquely near nonrefoldable sites (16.2%), which occurs at ~2-fold higher frequency than non-CASC sites being found uniquely near nonrefoldable sites (8.6%; Fig. 3F).

Although the proximity effect is statistically significant (*P* = 3.2 × 10^−6^ by Fisher’s exact test), we note that the effect size for this association (odds ratio = 2.04) is less pronounced than what we previously noted when considering entire proteins instead of peptides (odds ratio = 2.8; Fig. 2C). The interpretation of these findings is that while nonrefoldable proteins have a greater susceptibility to become structurally altered in cognitive impairment, the precise kinds of misfolded states that form in refolding experiments do not always correspond to the misfolded conformations associated with cognitive impairment. For instance, in LANCL1, the sites altered during refolding and cognitive decline are more diffuse and are not as close to one another.

### PTMs do not explain CASCs

**Fig. 4.**
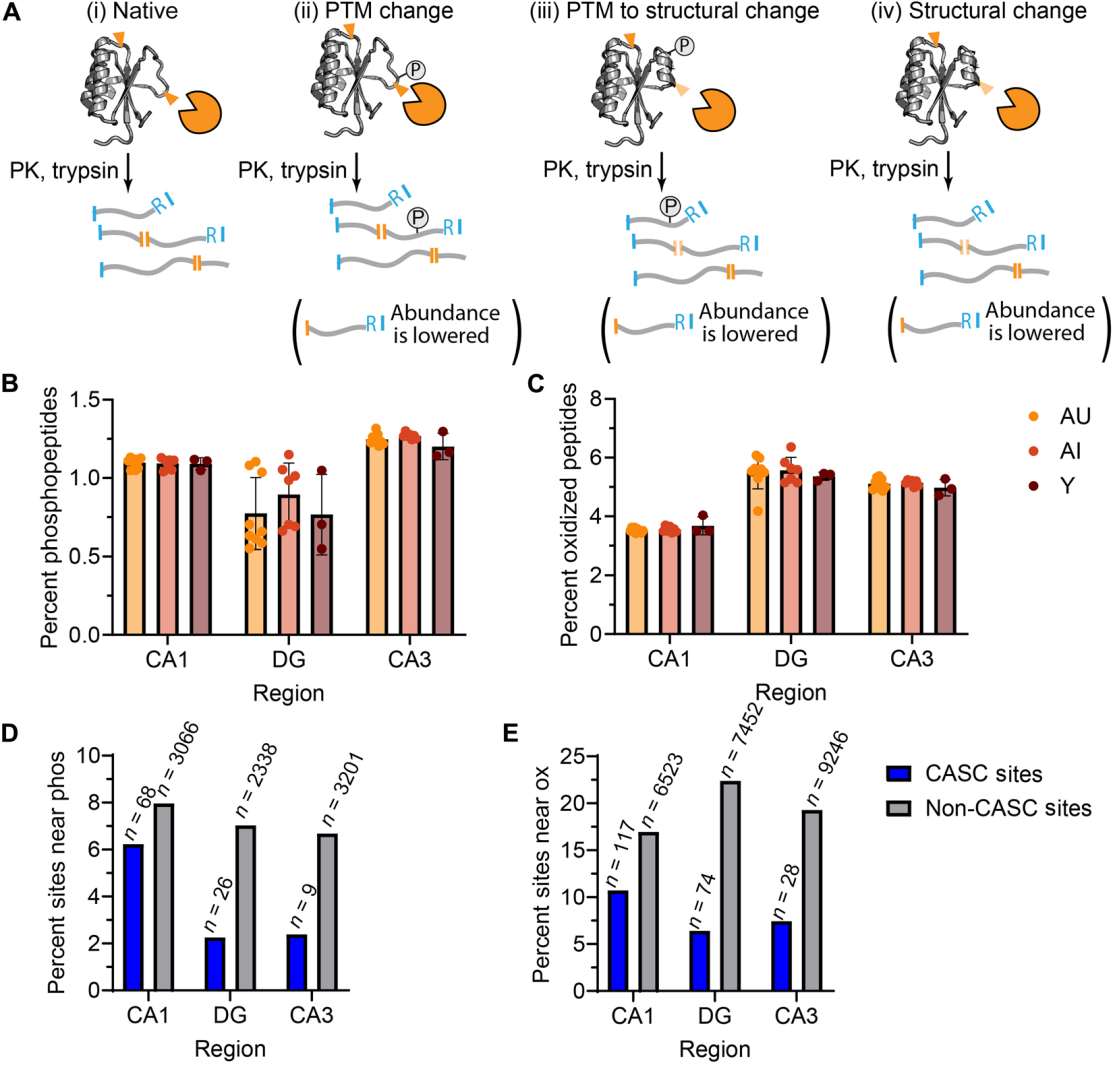
PTMs do not explain the preponderance of the cognition-based changes in proteolysis. (**A**) Scheme showing three scenarios (labeled cases ii, iii, and iv) for how the abundance of a half-tryptic peptide might be changed during LiP relative to a reference (case i). Case ii indicates a PTM directly changing a peptide abundance, since the mass of the peptide has changed with the modification; case iii indicates a PTM indirectly changing peptide abundance via an associated structural change; case iv indicates a change in proteolytic susceptibility only due to a structural (noncovalent) change. (**B** and **C**) Bar charts showing the percentage of total identified peptides (filtered to an FDR of 5%) that contain a phosphorylation (at Ser, Thr, or Tyr) (B) or oxidation (at Met, Phe, Tyr, or Trp) (C) from trypsin-digested peptides from hippocampal extracts associated with three subfields and three rat cohorts [young (Y), AI, and AU]. Dots represent individual biological replicates, bar heights represent averages, error bars represent SD. (**D** and **E**) Bar charts showing the percentage of peptides with significant cognition-associated changes (blue) and with no cognition-associated changes (gray) that are proximal to any detected phosphorylation site (D) or oxidation site (E). Proximity is within 15 residues on either side of the cut sites for half-tryptic peptides and within 15 residues on either side of the midpoint residue for tryptic peptides. Numbers above bars represent the total number of CASC and non-CASC sites within the given hippocampal region proximal to the PTM.

PTMs are commonly associated with aged proteins (*51*) and can also affect structure and function (*52*). Moreover, PTMs could potentially be confounding factors in our LiP experiment (Fig. 4A). First, the presence of a PTM could theoretically change the susceptibility of PK at a site near the modification (Fig. 4A, case iii), which could change the levels of half-tryptic peptide formed, resulting in a site being categorized as a CASC site, thereby acting as an indirect reporter of the change in PTM state between AU and AI subjects. In addition, the mass and ionizability of a peptide containing a PTM compared to a nonmodified peptide are different; hence, modification will decrease the abundance of a nonmodified peptide, but it would be incorrect to ascribe such a peptide abundance difference to a structural change when it is simply due to the existence of the PTM (Fig. 4A, case ii). We performed several analyses to test whether these effects could explain the cognition-associated changes in proteolytic susceptibility at hundreds of sites we identified. First, we re-searched all of the non-LiP raw data for phosphorylation modifications (+79.966 Da on Ser, Thr, and Tyr) and for oxidation modifications (+15.995 Da on Met, Phe, Tyr, and Trp). We could not find a meaningful difference in the frequency of phosphorylated peptides between AU, AI, and young rats within any region (CA1, DG, nor CA3) (Fig. 4B). We also could not see a difference in the frequency of peptides with oxidation modifications (Fig. 4C). While the hundreds of phosphosites and oxidation sites found are not comprehensive, they are sufficient to show the absence of a global difference between the test groups. Therefore, varying amounts of modifications between the groups (i.e., AU and AI) do not confound our LiP experiment, at least at the overall statistical level.

Next, we asked whether these modifications tended to be localized near PK cut sites, where they could potentially cause differences in PK susceptibility, which would be detected as a “significant” peptide by LiP-MS. Specifically, we looked at PK cut sites (or centers of full-tryptic peptides) on all CASC sites (and all non-CASC sites) and asked whether there were any detected phosphorylation or oxidation modifications located within 15 amino acids of the site. In general, we find that CASC sites are not close to PTMs, with 2 to 6% of CASC sites being close to detected phosphorylations and 5 to 10% of CASC sites being close to detected oxidations (Fig. 4, D and E, blue bars). We find a slight enrichment for non-CASC sites to be close to PTMs compared to CASC sites in all subfields of the hippocampus (Fig. 4, D and E, compare blue to gray bars). Hence, these findings are consistent with the notion that most of the differences in PK susceptibility that we encounter in this study are explained by differences in protein structure rather than by differences in PTM status (case iv in Fig. 4A). Moreover, these data show that PTMs do not overwhelmingly explain our CASC site identifications, as PTMs occur at similar frequencies both near CASC and non-CASC sites, and there is no major difference in PTM frequencies between AU, AI, and young within each hippocampal subfield.

### CASCs have diverse functions especially in the UPS

While we do not directly show that CASCs lead to functional changes, it stands to reason that, at least in some cases, these structural changes may be a cause or consequence of cognitive decline in aging as observed in the AI population. Structure is generally linked to function, so it is possible that some of these CASCs are dysfunctional in their soluble, yet structurally different, form. Structural changes could also be a result of different oligomerization states or differential binding to targets. Not only do we see specific CASCs with potential connections to cognition (see Fig. 3), but we also observe that some pathways contain many CASCs. The most notable example of this is the UPS.

**Fig. 5.**
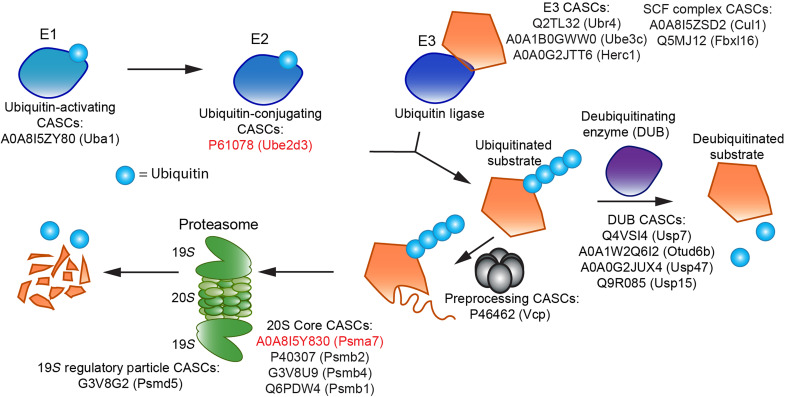
Many CASCs are part of the ubiquitin-proteasome pathway. UniProt accession numbers of various CASCs are listed, categorized by component in the UPS. Accession numbers highlighted in red are sCASCs. E1 activates ubiquitin. E2 conjugates ubiquitin to substrates that are recognized by an E3 enzyme. VCP is a protein that helps facilitate substrate degradation in some cases, such as by removing substrates from a membrane (*55*). The proteasome consists of a catalytic 20*S* core and 19*S* regulatory caps. DUBs remove ubiquitin from ubiquitinated proteins.

The UPS enables cell to degrade proteins that are no longer needed and is a highly regulated system of enzymes. Ubiquitin, a small protein that serves as a marker for degradation, is conjugated to protein substrates to be targeted for degradation. First E1 ubiquitin–activating enzymes conjugate to ubiquitin. The ubiquitin is then transferred to an E2 enzyme, which, in turn, transfers ubiquitin to a substrate that is specifically recognized by an E3 enzyme. The ubiquitinated protein is degraded by the proteasome, which is composed of a 20*S* catalytic core and 19*S* regulatory caps. Deubiquitinating enzymes (DUBs) serve as erasers for this modification to forestall the degradation of a ubiquitinated protein (*43*). CASCs are found in every step of this process (Fig. 5).

Of the 215 total CASCs, at least 17 CASCs are involved in the UPS. We identify an E1 CASC, an E2 CASC (also an sCASC; Fig. 3B), and three E3 CASCs. We also find two CASCs that are part of the SCF (Skp1–Cullin–F-box protein) complex. The SCF complex is an E3 complex, which consists of proteins Cullin 1 (Cul1), Rbx1, Skp1, and an F-box protein (*53*). Cul1 is a CASC, along with the F-box protein Fbxl16. Multiple components of the proteasome are also CASCs, including 26S proteasome non-ATPase regulatory subunit 5 (Psmd5) from the 19*S* cap, and four subunits from the 20*S* core [Psma7 (an sCASC), Psmb1, Psmb2, and Psmb4]. Psmb1 and Psmb2 (along with Psmb5, which is not a CASC) are catalytic subunits of the core, directly responsible for protein degradation (*54*). We also identified four unique DUBs as CASCs. We also find that Valosin-containing protein (VCP)/p97 is a CASC. VCP is an ATPase that extracts polyubiquitinated proteins from membranes, organelles, and complexes; unfolds them to facilitate their degradation; and plays a key role in the endoplasmic reticulum–associated degradation pathway (*55*).

The pervasive appearance of CASCs at every stage of the UPS suggests that there may be some dysfunction in this system. It is already known that protein turnover is impaired with age (*56*), and proteasome activity has been shown to be decreased in the aged brain (*57*). Since our study compares two aged populations, we posit that there could be a difference in proteasome function between different cognitive phenotypes, which would need to be investigated further.

We also see many CASC proteins involved at the synapse. Synapses are often affected by aging (*58*, *59*) and are critical for memory formation and long-term potentiation (*60*). Gephyrin plays a critical scaffolding role at the synapse of inhibitory neurons (*61*) and is a CASC protein. Some work has suggested that neuronal hyperactivity may be detrimental to the aging brain, as neuronal inhibition in *Caenorhabditis elegans* was shown to increase lifespan (*62*), and cognitively impaired rats have hippocampal overactivity compared to age-matched unimpaired rats (*63*). Further structural and functional characterization of this protein at the synapse may help elucidate the molecular basis for neuronal overactivity and its linkage to cognitive decline.

14-3-3 proteins are enriched at synapses as well (*64*) and are involved in regulating levels of hippocampal postsynaptic *N*-methyl-d-aspartate (NMDA) receptors (*65*), which are important for synaptic plasticity. In addition, lower NMDA receptor activity has been suggested to lead to neuronal hyperactivation (*66*), which is a phenotype seen in AI rats (*63*). 14-3-3 proteins, in general, have also been identified as having potential molecular chaperone–like activities (*67*). We find that both 14-3-3 protein γ and 14-3-3 protein β/α are both CASCs. We identify other CASC proteins that function at the synapse, including neurobeachin, syntaxin binding protein 5, and synapsin II.

We also identified many CASCs involved in translation. For example, elongation factor 2 (EF2) is a CASC and helps translocate the ribosome during translation. Several aminoacyl tRNA synthetases, such as those for methionine (A0A8I5ZKR4), glutamine (Q66H61), and isoleucine (A0A0G2K261), were identified as CASCs, along with other proteins that act on tRNAs such as tRNA N^6^-adenosine threonylcarbamoyltransferase (Q9WVS2) and peptidyl-tRNA hydrolase (D3ZBG6), the latter of which is important for rescuing stalled ribosomes (*68*). Initiation factors 4A-I (Q6P3V8), 4A-II (Q5RKI1) and 3 subunit B (Q4G061) are also CASCs. Two components of the TRiC (TCP-1 ring complex) chaperonin (T-complex protein 1 subunit theta (Cct8) and T-complex protein 1 subunit alpha (Tcp1), D4ACB8 and P28480) are also identified as CASCs. The TRiC complex is a chaperone, which is required for the correct folding of some proteins, especially tubulins (*69*). CASCs are found in several places in the ribosomal translation system, indicating that there may be structural changes affecting translation between the cognitive phenotypes. Since translation is the first opportunity for a protein to fold correctly, this could have broad implications for how proteins become misfolded, and dysfunctional translation could potentially contribute to the formation of other CASCs. In addition, translation has been shown to change with age [for example, EF2 has lower activity with age (*70*), and ribosomal pausing is increased (*71*)]; perhaps, structural changes in EF2 and other translation factors may contribute to these perturbations.

We wondered whether specific pathways were enriched in the set of nonrefoldable proteins. While we were not able to conduct a formal gene ontology (GO) analysis on CASCs due to the low number of CASCs, we used ShinyGo (*72*) to perform GO analyses for the nonrefoldable protein set. GO analysis using the Kyoto Encyclopedia of Genes and Genomes (KEGG) database (*73*) shows that nonrefoldable proteins are enriched in categories relating to metabolism, the proteasome, and neurodegenerative diseases (fig. S7A). Cellular component GO showed enrichment of nonrefoldable proteins in the proteasome and related structures and the proton-transporting V-type ATPase complex (fig. S7B). Analysis using the Rat Genome Database (RGD) GO terms (*74*) shows that nonrefoldable proteins are overenriched in metabolic pathways (namely, glycolysis, gluconeogenesis, and the pentose phosphate pathway) and the ubiquitin/protease degradation pathway (fig. S7C). Notably, the GO analyses for the nonrefoldability data show that at least some categories that appear to be enriched in CASCs (such as the UPS) are also overenriched among nonrefolders. This further strengthens the finding that CASCs may be more likely to occur if a protein is nonrefoldable.

## DISCUSSION

Here, we have used LiP-MS to identify proteins that vary in structure in the hippocampus of aged rats with or without cognitive impairment, which we have defined as CASC proteins. We identified 215 CASC proteins in the CA1 hippocampal region. Research in aging, dementia, and neurodegenerative disease has long made a connection between these disease processes and protein misfolding; however, emphasis has historically been paid to proteins that form amyloids or other insoluble aggregates. We have focused on the soluble fraction of the hippocampal proteome and used a methodology that can sensitively detect subtle changes in protein structure. The results enable us to conclude that protein misfolding is perhaps a more pervasive feature in cognitive decline than previously appreciated and that many of these misfolded forms persist as soluble species. This finding suggests that there may be previously unidentified avenues for potential therapeutic targets and diagnostic biomarkers for cognitive decline than the small subset of amyloid-forming proteins frequently studied. Of course, these interventions would need to be conformation specific, creating additional opportunities and challenges.

Strictly speaking, the changes in proteolytic susceptibility that we detect (Fig. 1) could be explained by a wide range of factors, including changes in interaction partners, changes in oligomeric stage, changes in protein localization, and changes in PTMs. One of the limitations of our study is that we cannot directly identify the exact physical or functional nature of structural changes that occur in CASCs. As proteins age, they can accumulate PTMs, including enzymatic PTMs such as phosphorylation and nonenzymatic PTMs such as oxidation (*51*). These PTMs can affect the folding and the function the proteins they modify (*52*) and can also cause conformational changes, even at regions of the protein far from the PTM site itself (*75*). Our results suggest that most of the changes in proteolytic susceptibility observed in our assay can be ascribed to changes in conformation or misfolding. We were able to rule out the possibility that PTMs explain the majority of the signal because they can also be localized by MS, and we could not find evidence for strong colocalization to CASC sites. While it is likely that some of the CASCs we identify report on changes in interaction partners or oligomeric state, the strong overlap between CASC status and nonrefoldability (Fig. 2C) provides indirect evidence that these features arise from a common underlying etiology in a protein being complicated to fold or prone to misfold.

Our refoldability experiments use a frequent condition used in classic protein folding studies, namely, complete denaturation in 6 M GdmCl, followed by rapid dilution to renaturing conditions. These conditions do not mimic any normal physiological stressor and certainly not the slow progressive changes associated with aging. Nevertheless, we would argue that refoldability under these conditions is relevant because it reports on the ability of a protein to navigate to its native structure spontaneously and unassisted. Refoldable proteins can be viewed as potentially intrinsically more resistant to age-related structural changes because no matter what types or magnitudes of stressors they may experience that temporarily result in unfolding or misfolding, they have the inherent capacity to fold to their native structure autonomously. On the other hand, nonrefoldable proteins are more likely to be unable to recover from the damage accrued over time because they cannot efficiently self-correct them; hence, they are more likely to accumulate these deformations over age in the context of a disrupted proteostasis network. In addition, while conventional thinking about the timescales associated with protein folding might assume that only covalent modifications would represent an accumulable form of damage over longer timescales, recent work on protein entanglement has demonstrated that certain misfolded conformations having changes in entanglement can be long lived (*76*) and also correlate with nonrefoldability (*10*).

We note that the precise way proteins misfold upon attempted refolding from denaturant versus during aging are likely distinct in many cases. The association between CASC and nonrefoldability when looking at peptides specifically was not as robust as when considering entire proteins, as CASC sites are only approximately twofold more likely to be located near sites with altered structure after refolding. Although there are a few interesting exceptions (such as Atp6v0c; compare Fig. 3), this negative result is logical, as age-related protein misfolding would not be expected to occur from a globally denatured state, as it does in our in vitro refolding study. On the other hand, the data presented here present a case that nonrefoldable proteins, because of their incapacity to self-correct, may be more likely to be permanently affected by stressors such as aging and hence provide a unique window into the biophysics behind aging and proteostasis loss.

This study shows that soluble proteins with conformational differences exist between the hippocampus of rats with age-related spatial learning and memory impairment and those without, but at this stage, does not yet provide a mechanism for how these altered conformations form or persist. It is important to point out that CASC proteins are not amyloids. Because of their stability, amyloids are resistant to many degradation pathways (*77*), providing a rationale for why they can persist and accumulate over time during aging. However, using a bioinformatic predictor for amyloid formation propensity (*78*), we find that CASC proteins are not, in general, more likely to be amyloidogenic than nonamyloidogenic (fig. S8). Hence, an alternative model is required.

We put forward three potential explanations for how soluble misfolded conformations might populate and note at this stage that these are hypotheses that would need to be explored deeper with future study. These explanations are all related to proteostasis. One possibility is that CASC proteins are those that are more reliant on various molecular chaperones to properly fold. Rats with cognitive decline may have lower levels of certain chaperones or less functional chaperones, resulting in CASC proteins (which often contain complicated structural elements) becoming the first “victims” to an impaired proteostasis network. Some chaperones have been found at lower levels in aged cells (*79*, *80*), and chaperone function has been linked to cognition (*81*). In addition, the chaperones heat shock protein 90β (Hsp90β) (P34058) and the Hsp70s HSPA4 and HSPA12A (O88600 and D3ZC55) are themselves CASCs, meaning that they may not be functioning identically between cognitive phenotypes. Potentially, a structurally altered chaperone may not be able to properly assist with protein folding, begetting more CASCs. However, another possibility is that the chaperones are present in different functional states because their activity is affected by the presence of other CASCs. For example, heat shock proteins show strong LiP signals during and after heat shock in yeast (*38*). In general, but particularly for chaperones, LiP-MS cannot discriminate between a misfolded protein conformation and an alternative functional conformation.

A second possibility is that CASC proteins are those that are more dependent on cotranslational folding to fold. Recent studies have suggested that translation can be dysregulated in aged cells (*71*, *82*) and this could provide a global stress that is particularly acute for proteins sensitive to the precise timing of translation elongation or that require chaperones, which act cotranslationally (*83*). Circumstantial evidence supporting this view is that CASC proteins tend to be nonrefoldable, which may be more reliant on cotranslational folding to bypass incorrect kinetically trapped conformations (*39*). We also identified eukaryotic EF2 as a CASC. EF2 is essential for protein synthesis, and lower activity has been demonstrated with age (*70*), suggesting a possible age-related structural change. A structural change in such a critical protein for translation may have a functional effect on translation in vivo. In addition, we detected several other translation-related proteins as CASCs including aminoacyl tRNA synthetases and tRNA-modifying enzymes, which could alter elongation kinetics and therefore proper protein folding.

A third possibility is that CASC proteins represent conformations with impaired turnover; hence, despite an altered non-native structure, they are degraded more slowly than their correctly folded native forms, which results in a slow accumulation over the course of time. Protein clearance mechanisms, such as autophagy, are already known to be impaired with age (*15*), along with protein turnover in general (*56*). In support of this hypothesis, when we cross-compare our results to their mouse orthologs in a proteome-wide assessment of protein lifetimes in the mouse brain (*84*), we find a strong association wherein long lifetime proteins have a high propensity to be CASC (e.g., proteins with lifetimes of >32 days are 19% CASC), and short lifetime proteins are less likely to be CASC (e.g., proteins with lifetimes of <4 days are 6% CASC; 21-month-old mouse cortical proteins; fig. S9). The trend is present when considering lifetimes of proteins from cortex homogenates or synaptic fractions or proteins from old mice (aged 21 months) or young mice (aged 5 months; fig. S9). This finding suggests that low lifetime proteins may be degraded before they have an opportunity to populate aberrant conformations, while proteins that persist for longer are at greater risk. Although protein turnover in the brain has been studied, previous experiments have not yet explored conformation-specific changes in turnover, which could prove to be an interesting area for future study. One hypothesis is that longer lifetime proteins might undergo misfolding events that further perturb their turnover, which would, in turn, provide a way for them to accumulate slowly over the course of aging. We caution, however, that the present study cannot yet establish whether the subpopulation of proteins that populate CASC conformations are older or newly synthesized.

A further caveat is that the subfield dissection approach used in the current study does not allow for definitive identification of where proteins are produced, only where they are localized. For example, the CA1 molecular layer would include axon terminals from CA3 Schaffer collaterals and from entorhinal cortex inputs. This leaves open the possibility that CASCs identified in the CA1 preparation could have originated from upstream synaptic partners. If CASCs formed primarily at synapses, then the CASCs identified in the CA1 dissection could have originated from cell bodies of CA3 neurons. Previous studies identified greater changes in transcription and neuronal hyperactivity in CA3 in AI rats (*6*, *25*, *29*, *63*); further research will be needed to better elucidate if changes in protein structure in CA1 are connected to the other changes observed in CA3 neurons.

To summarize, we have found that structural proteomics is an emerging “omics” method that is particularly relevant for dissecting the features associated with progressive age-related cognition loss. We identified proteins that differ in structure between two cognitive phenotypes in aging and showed that this group is enriched with proteins that cannot spontaneously refold after denaturation. Modern methods such as LiP-MS are appropriately sensitive to detect subtle structural alterations in soluble misfolded proteins, which are pervasive in aged rats’ hippocampi. Further mechanistic and structural studies on specific CASC proteins could promote some of the proteins disclosed here to be considered therapeutic targets or early disease biomarkers.

### Limitations of the study

Because LiP signals can arise from a number of factors, the study would benefit from an orthogonal structural proteomic assay for validation. Current methods include thermal proteome profiling (TPP) (*85*), cross-linking MS (XL-MS) (*86*), fast photochemical oxidation of proteins (FPOP) (*87*), stability of proteins from rates of oxidation (SPROX) (*88*), and covalent protein painting (CPP) (*89*). TPP and SPROX do not report on conformational changes per se, and so overlap with CASCs would not necessarily be expected, and XL-MS typically does not work as well for samples with proteome-wide complexity. On the other hand, CPP and FPOP could serve as meaningful comparisons. Ultimately, before a CASC protein should be considered a therapeutic target, detailed structural characterization using a method such as cryo–electron microscopy would be necessary. In addition, functional assays on proteins extracted from AU and AI hippocampi would be important to test whether the structural changes detected by LiP-MS ultimately affect the protein’s activity.

LiP-MS, similar to many structural proteomic methods, is biased in favor of providing more data on abundant proteins. While the statistical procedures used here mitigate for labeling proteins CASC purely because they have greater coverage, structural changes in low-abundance proteins are still challenging to detect. We have found that applying structural proteomics to a complex phenotype such as age-related cognitive decline creates additional challenges, such as higher levels of intragroup variability and missing data. In addition, since the rat model itself is genetically variable, high variability is expected (fig. S3). These features result in higher FDR, which we estimate through permutation analyses and which we would recommend for future studies. Some of these issues may be mitigated by data-independent acquisition methods, which tend to lead to fewer missing data for many precursor ions and for which LiP-compatible methods have recently been developed (*90*).

Last, the study does not prove that the structural changes observed are causal for cognitive decline. Although the concentration of CASCs in proteostasis pathways is suggestive, detailed mechanistic study would be needed to establish a causal linkage between CASCs and cognitive phenotype. While structure is, in general, related to function, this method does not report on if and how these structural changes may relate to function and if these changes are causative of, a result of, or unrelated to the differences in cognition between these two populations.

## MATERIALS AND METHODS

### Animal care and tissue collection

Long-Evans rats were obtained from Charles River Laboratories. All rats are male. Aged (AU and AI) rats were obtained as retired breeders at ~6 to 9 months of age and housed in a vivarium at Johns Hopkins University until 24 months of age. Young rats were also obtained from Charles River Laboratories at ~4 to 6 months of age and housed in the same vivarium. Food and water were given ad libitum, and animals were on a 12-hour light/dark cycle. Animals housing and use were performed in accordance with protocols approved by the Johns Hopkins University Institutional Animal Care and Use Committee, protocol number RA22A402.

Twenty animals were used for the CA1 CASC LiP experiment (10 AU, 7 AI, and 3 young). The DG and CA3 studies used the same animals, except omitting one AU animal for a total of nine AU, seven AI, and three young. Two weeks after completion of behavioral testing, all rats were deeply anesthetized with isoflurane and euthanized by rapid decapitation. The 2-week time frame was chosen to maximize the likelihood of detecting stable, baseline protein profile differences, and to minimize the effects of learning- or swimming-induced differences. Brains were immediately extracted and placed in ice-cold phosphate-buffered saline. The hippocampus was separated from the brain, and for the CASC LiP study, the CA1, CA3, and DG were microdissected by hand from 400-μm transverse sections of the hippocampus along its entire longitudinal extent. Hippocampi for the refoldability study were not subfield dissected (*91*). Tissue was snap frozen on dry ice and moved to a −80°C freezer for storage.

### MWM cognitive assessment

Behavioral assessment of memory function in an MWM task was conducted as previously described (*31*). Briefly, rats were trained for 8 days (three trials per day) to locate a camouflaged escape platform that remained at the same location throughout training in a water maze. Every sixth trial consisted of a probe trial (no escape platform for the first 30 s of the trial) that served to assess the development of a spatially localized search. Learning index scores were derived from each rat’s proximity to the platform during probe trials, with lower scores reflecting a more accurate search indicating better retention of the platform location. A learning index cutoff of 240 was used to identify aged rats as AU or AI, with higher scores representing worse performance and reflecting scores that fall inside or outside the normative range collected from young adult Long-Evans rats over many years. Cue training, where the platform was visible after completion of the hidden platform tests, was used to assess the sensorimotor and motivational status of the rats. Only rats with successful cue training performance were included in the present study.

### Aging/cognition structural proteomics experiment

#### Limited proteolysis

Frozen hippocampal subfields were removed from the −80°C freezer and immediately added to 1 ml of chilled lysis buffer [20 mM tris (pH 8), 150 mM KCl, 10 mM NaCl, and 2 mM MgCl_2_] containing protease inhibitors [final concentrations of 0.5 mM phenylmethylsulfonyl fluoride (PMSF), 0.05 mM bestatin, and 0.015 mM E64, all diluted from 100× stock in dimethyl sulfoxide (DMSO)] and deoxyribonuclease (DNase) [final concentration of 0.1 mg/ml, diluted from 100× stock in Millipore water (MPW)] in a 1-ml Dounce homogenizer (Wheaton, USA) on ice. Samples were vigorously homogenized on ice. Samples were processed in a mixed order by phenotype to avoid bias. Homogenized tissue was moved to a 1.5-ml microfuge tube and clarified at 15,000*g* for 15 min at 4°C to remove insoluble cellular debris. The supernatant was moved to a fresh 1.5-ml microfuge tube and allowed to sit at room temp for at least 2 hours and 20 min before LiP to ensure that PMSF had sufficient time to hydrolyze (approximately four half-lives) and would not inhibit our protease during the LiP step. During this time, protein concentrations of the samples were determined using the bicinchoninic acid (BCA) assay, according to the manufacturer’s protocol (Pierce Rapid Gold Protein Assay Kit, Thermo Fisher Scientific, A53225) and using bovine serum albumin (BSA) as a standard. Lysates were normalized to 1 or 0.5 mg/ml by dilution with lysis buffer.

After adequate time for hydrolysis of PMSF has passed, LiP can be performed. PK [Thermo Fisher Scientific, 17916; previously prepared as stock (1 mg/ml) in 1:1 (v/v) of lysis buffer to 20% glycerol, aliquoted, flash-frozen, and stored at −20°C] was diluted such that 2 μl of the dilution would contain 1:100 (w/w) of PK to the amount of sample to be digested (in this case, either 1 or 2 μg for 100 or 200 μg of sample, respectively). Two microliters of appropriately diluted PK was placed at the bottom of a fresh 1.5-ml microfuge tube. Normalized sample (200 μl) was added to the tube containing 2 μl of PK and pipetted rapidly up and down seven times to mix thoroughly. This mixture was incubated for exactly 1 min before the tube was added to a 105°C mineral oil bath for 5 min to quench the PK reaction. For each sample, a corresponding no-PK, non-LiP control was performed. In this case, 200 μl of normalized lysate was added to a fresh 1.5-ml tube without PK and then added to the oil bath for 5 min. After 5 min, all samples were removed from the oil bath and quickly centrifuged to collect condensation and transferred to a new 2-ml tube containing 152 mg of urea (final concentration of 8 M urea and 314 μl final volume).

#### MS sample preparation

To each sample in 8 M urea, to reduce disulfides, DTT was added to a final concentration of 10 mM (4.5 μl added from a freshly made 700 mM DTT stock) and the samples were incubated in a thermomixer for 30 min at 37°C and 700 rpm. To cap cysteines, iodoacetamide (IAA) was added to a final concentration of 40 mM (18 μl added from a freshly made 700 mM IAA stock) and the samples were incubated at room temperature in the dark for 45 min. Samples were diluted to a final concentration of 2 M urea by adding 1010 μl of a freshly made 100 mM ammonium bicarbonate (ambic) stock. To each sample, 2 μg of trypsin (New England Biolabs) was added [1:50 (w/w) of trypsin to protein]. Samples were incubated overnight (~16 hours) in a thermomixer at 25°C and 700 rpm. Digested samples were acidified with trifluoroacetic acid (TFA; Acros) to a final concentration of 1% by volume by adding 16.6 μl of TFA. Peptides were desalted using Sep-Pak Vac 1 cc (50 mg) C18 cartridges (Waters). Cartridges were placed on a vacuum manifold and conditioned by adding 2× 1 ml of buffer B [0.5% TFA in 80% LC grade acetonitrile (ACN) and 20% LC grade water]. Cartridges were equilibrated with 4× 1 ml of buffer A (0.5% TFA in LC grade water). Peptide samples were loaded on each cartridge. Cartridges were washed with 4× 1 ml of buffer A. Cartridges were removed from the vacuum manifold, placed in a 15-ml conical tube, and eluted by adding 1 ml of buffer B to the cartridge and spinning in a swing bucket rotor (Eppendorf 5910 R) at 300 rpm for 5 min. Eluent was transferred from the conical vial to a fresh 1.5-ml microfuge tube and dried using a vacuum centrifuge (Eppendorf Vacufuge Plus). Dried peptides were stored at −80°C until ready for analysis.

#### MS data acquisition

Peptide samples were resuspended in 0.1% formic acid (Optima, Fisher Scientific) in LC grade water (Optima, Thermo Fisher Scientific) to a final concentration of 1 μg/μl. Approximately 1 μg of peptides were injected into a Thermo Fisher Scientific UltiMate 3000 UHPLC system for chromatographic separation. The column temperature was maintained at 40°C, and the flow rate was 0.300 μl/min for the duration of the run. Solvent A [0.1% formic acid (FA)] and solvent B (0.1% FA in ACN) were used as the chromatography solvents. Peptides were allowed to accumulate onto the trap column (Acclaim PepMap 100, C18, 75 μm by 2 cm, 3 μm, 100-Å column) for 10 min (during which the column was held at 2% solvent B). The peptides were resolved by switching the trap column to be in line with the separating column (Acclaim PepMap RSLC, C18, 75 μm by 2 cm, 2 μm, 100-Å column), increasing the gradient to 5% solvent B over 5 min and then applying a 95-min linear gradient from 5 to 25% solvent B. Then, the gradient was increased linearly to 40% solvent B over 25 min. Next, the gradient was held at 40% solvent B for 5 min and then increased again from 40 to 90% solvent B. The gradient was held at 90% solvent B for 5 min. The column was then cleaned with a sawtooth gradient to purge residual peptides between runs in a sequence. A 1-hour blank cleaning sequence was also run between every three and six samples to ensure a clean column.

A Thermo Fisher Scientific Q Exactive HF-X Orbitrap mass spectrometer was used to analyze protein digests. A full MS scan in positive ion mode was followed by 20 data-dependent MS scans. The full MS scan was collected using a resolution of 120,000 [at mass/charge ratio (*m*/z) of 200], an AGC target of 3 × 10^6^, a maximum injection time of 64 ms, and a scan range from 350 to 1500 *m*/*z*. The data-dependent scans were collected with a resolution of 15,000 (at *m*/z of 200), an AGC target of 1 × 10^5^, a minimum AGC target of 8 × 10^3^, a maximum ion injection time of 55 ms, and an isolation window of 1.4 *m*/*z* units. To dissociate precursors before their reanalysis by MS2, peptides were subjected to a higher-energy collisional dissociation (HCD) of 27% normalized collision energies. Fragments with charges of 1, 6, or higher and unassigned were excluded from analysis, and a dynamic exclusion window of 30.0 s was used for the data-dependent scans.

#### Data analysis: LFQ and CASC determination

For analysis of the data, FragPipe (version 22.0) was used. Two LFQ match between run (LFQ-MBR) analyses were run for each subfield—one with all the no-PK (trypsin-only) control samples and one with the LiP samples (which contain PK). MSFragger (version 4.1) and IonQuant (version 1.10.27) were used for peptide identification and quantification within FragPipe. We also ran three null analyses per region, where samples were randomly assigned AU and AI regardless of actual cognitive status to assess the rate of false positives. Default LFQ-MBR settings were used, except where specified otherwise. The data were searched against the FASTA reference proteome for rats, *Rattus norvegicus* (UP000002494, UniProt.org) with decoys and contaminants added by FragPipe. Samples were given the “experiment” label of AU, AI, or young. In MSFragger, precursor mass tolerance was set to −10 to 10 parts per million, and a semispecific strict trypsin searches were performed, with up to two missed cleavages allowed. Peptide lengths of 7 to 50 and masses of 500 to 5000 were permitted. Methionine oxidation, clipped N terminus, and N-terminal acetylation were set as dynamic modifications, and cysteine carbidomethylation was set as a static modification. Output format was selected to be TSV_PEPXML_PIN. IonQuant MBR ion FDR was set to 0.05, and the MBR top runs parameter was set to 100.

For analysis of individual samples, the peptide.tsv and protein.tsv files from each individual sample were analyzed to gather quality metrics such as number of peptides, number of proteins, and percent half-trypticity. In-house Python (version 3.11) code counted the number of proteins with a ProteinProphet score greater than 0.95 from the protein.tsv file, counted the number of peptides with a PeptideProphet score greater than 0.95 from the peptide.tsv file, and determined the percentage of those peptides that were half-tryptic. Half-tryptic peptides are peptides with one end immediately preceding arginine or lysine (the two amino acids after which trypsin cuts). Fully tryptic peptides have both ends immediately preceded by arginine or lysine.

For analysis of the LFQs, we used a modified version of the tool FLiPPR (*34*). FLiPPR uses the FragPipe combined_ion.tsv that is generated by FragPipe for both the control and LiP LFQs to identify significantly different peptides between conditions, normalize LiP peptide abundances (if needed) based on the control LFQ, implement a Benjamini-Hochberg FDR correction per protein, and output the number of valid peptides and the number of significantly different peptides per protein. Modifications to FLiPPR were necessary to accommodate for differing number of replicates under each condition (i.e., 10 AU and 7 AI) and to provide greater flexibility for imputing missing values and filtering ions based on the amount of missing data.

Two different analyses were performed per region, first, a strict analysis that did not include any imputed values for missing data was performed. In this case, FLiPPR used the original combined_ion.tsv generated from FragPipe as input. For the general analysis, imputed values for missing data were used. Several conditions were tested to find conditions that were not too permissive, leading to false positives due to the variable nature of the data, but not so restrictive that we filter many significant peptides (table S3). In LiP, missing data can be expected frequently, as PK can generate different peptides between different conditions if there are true structural changes between proteins. Because of this, we feel that it is appropriate to impute values for missing data in some cases, namely, where one category (such as AU) is not missing many values, while the other category (such as AI) is missing many values. For imputation of missing values, we used an in-house Python script to identify peptides where >66% of data are missing under one condition but, under the other condition, >55% of data are present. Missing values were then replaced with a random value taken from a Gaussian distribution centered around 10,000 with an SD of 1000 for the condition with more than 66% missing data. This new file with imputed values was then used as input for the modified FLiPPR code. The same modified FLiPPR code was used for both the strict and general analyses, but the strict analysis inputs the original combined_ion.tsv FragPipe output, and the general analysis inputs the same file but with the imputed values.

Details regarding FLiPPR’s algorithm can be found in Manriquez-Sandoval *et al.* (*34*). In the modified code, each feature from the combined_ion file was considered. If there were more than five values missing from AU and more than three values missing from AI (four and three, respectively, for DG and CA3 due to the lower number of AU replicates), then the feature was filtered out. In the case where a feature is not filtered out but still contains missing values, these missing values are dropped, but the rest are retained. Features in the combined ion file are merged to peptides by taking the median of the ratios of AU/AI ions, and *P* values are combined using Fisher’s method if the ratios agree in direction. Otherwise, the *P* value is set to one. Normalization of the abundance of a peptide from the LiP experiment occurs if the corresponding peptide in the control experiment has a fold change greater than twofold and a *P* value (using Welch’s correction for unequal population variances) less than 0.01.

FLiPPR outputs a summary file with a list of proteins, the number of valid peptides, and the number of significant peptides. A peptide is considered significant if the abundance change is greater than twofold between AU and AI (reported in log_2_) and if the adjusted *P* value (using Welch’s correction for unequal population variances and the Benjamini-Hochberg method for multiple hypothesis correction) is less than 0.05 (reported as −log_10_). The Benjamini-Hochberg correction is applied on a per-protein basis, since, for LiP experiments, the null hypothesis is that a protein is not structurally different. Each peptide provides data to potentially reject this hypothesis. Applying the Benjamini-Hochberg correction on a per-protein basis ensures that proteins that have more peptides/higher coverage are not considered structurally perturbed simply because there were more chances to disprove the null hypothesis.

If a protein has two or more significantly different peptides, then it is considered a protein with a CASC. The protein is considered an sCASC if it has two or more significantly different peptides in the strict analysis.

### Refoldability assay

#### Preparation of native and refolded hippocampal extracts

Three frozen whole hippocampi from young (~6-month-old) rats were removed from the −80°C freezer and each immediately added to 1 ml of chilled lysis buffer [20 mM tris (pH 8), 100 mM NaCl, and 2 mM MgCl_2_] containing protease inhibitors (final concentrations of 0.5 mM PMSF, 0.05 mM bestatin, and 0.015 mM E64, all diluted from 100× stock in DMSO) and DNase (final concentration of 0.1 mg/ml, diluted from 100× stock in MPW) in a 1-ml Dounce homogenizer (Wheaton, USA) on ice. Samples were vigorously homogenized on ice. Homogenized tissue was moved to a 1.5-ml microfuge tube and clarified at 16,000*g* for 15 min at 4°C to remove insoluble cellular debris. The supernatant was moved to a fresh 1.5-ml microfuge tube and kept on ice. Protein concentrations of the samples were determined using the BCA assay, according to the manufacturer’s protocol (Pierce Rapid Gold Protein Assay Kit, Thermo Fisher Scientific, A53225) and using BSA as a standard. Lysates were normalized to 2 mg/ml by dilution with lysis buffer.

To prepare native samples, normalized lysate was diluted to a final concentration of 0.115 mg/ml using native dilution buffer [20 mM tris (pH 8), 100 mM NaCl, 2 mM MgCl_2_, 1.061 mM DTT, and 0.0637 M GdmCl] by adding 11.5 μl of lysate (2 mg/ml) to 188.5 μl of native dilution buffer. This buffer has a small amount of GdmCl to match the concentration of GdmCl that will be present after refolding of unfolded samples. Final concentrations of all components were protein (0.115 mg/ml), 20 mM tris (pH 8), 100 mM NaCl, 2 mM MgCl_2_, 0.06 mM GdmCl, and 1 mM DTT. Native samples were incubated at room temperature for 1 hour before proceeding to LiP. A total of 2.5 hours must have passed since initial addition of PMSF to ensure that it is hydrolyzed sufficiently to not interfere with PK in the LiP step.

To prepare refolding samples, proteins were first unfolded. Initially, 500 μg of protein [250 μl of lysate (2 mg/ml)] was added to a fresh 1.5-ml microfuge tube, and 25 mg of GdmCl powder and 0.61 μl of 700 mM DTT stock were added. Refolding samples were placed in a Vacufuge Plus (Eppendorf), and volume was reduced to 43 μl. Final concentrations of all components were protein (11.5 mg/ml), 115 mM tris (pH 8), 575 mM NaCl, 11.5 mM MgCl_2_, 6 M GdmCl, and 10 mM DTT. Samples were incubated at room temperature overnight to ensure complete unfolding of the samples.

To refold the samples, samples were diluted 100× in refolding dilution buffer [19.03 mM tris (pH 8), 95.14 mM NaCl, 1.9 mM MgCl_2_, and 0.909 mM DTT] by adding 10 μl of unfolded sample into 990 μl of refolding dilution buffer. Final concentrations of all components were protein (0.115 mg/ml), 20 mM tris (pH 8), 100 mM NaCl, 2 mM MgCl_2_, 0.06 mM GdmCl, and 1 mM DTT. All concentrations are the same as the native samples. Refolded samples were incubated for 1 min, 5 min, or 2 hours at room temperature to allow proteins to refold before proceeding to the LiP step.

#### Limited proteolysis

After the prescribed time had passed (1 min, 5 min, or 2 hours from refolding for refolded samples, and 1 hour from native dilution for native samples), LiP was performed. PK [Thermo Fisher Scientific, 17916; previously prepared as stock (1 mg/ml) in 1:1 (v/v) of lysis buffer to 20% glycerol, aliquoted, flash-frozen, and stored at −20°C] was diluted, such that 2 μl of the dilution would contain 1:100 (w/w) of PK to the amount of sample to be digested (in this case, 0.23 for 23 μg of sample). Two microliters of appropriately diluted PK (0.115 mg/ml) was placed at the bottom of a fresh 1.5-ml microfuge tube. Sample (200 μl) was added to the tube containing 2 μl of PK and mixed by a quick vortex, followed by a short spin in a benchtop centrifuge to ensure that all sample is at the bottom of the tube. This mixture was incubated for exactly 1 min (including vortex and centrifugation time) before the tube was added to a 105°C mineral oil bath for 5 min to quench the PK reaction. For each native sample and 2-hour refolding sample, a corresponding no-PK, non-LiP control was performed (six total). In this case, 200 μl of normalized lysate was added to a fresh 1.5-ml tube without PK and then added to the oil bath for 5 min. After 5 min, all samples were removed from the oil bath and quickly centrifuged to collect condensation and transferred to a new 2-ml tube containing 152 mg of urea (final concentration of 8 M urea and 314 μl f.v.).

#### MS sample preparation

To each sample in 8 M urea, to reduce disulfides, DTT was added to a final concentration of 10 mM (4.5 μl added from a freshly made 700 mM DTT stock) and the samples were incubated in a thermomixer for 30 min at 37°C and 700 rpm. To cap cysteines, IAA was added to a final concentration of 40 mM (18 μl added from a freshly made 700 mM IAA stock) and the samples were incubated at room temperature in the dark for 45 min. Samples were diluted to a final concentration of 2 M urea by adding 942 μl of a freshly made 100 mM ambic stock. To each sample, 0.46 μg of trypsin (New England Biolabs) was added (1:50 (w/w) of trypsin to protein). Samples were incubated overnight (~16 hours) in a thermomixer at 25°C and 700 rpm. Digested samples were acidified with TFA (Acros) to a final concentration of 1% by volume by adding 12.8 μl of TFA. Peptides were desalted using Sep-Pak Vac 1 cc (50 mg) C18 cartridges (Waters). Cartridges were placed on a vacuum manifold and conditioned by adding 2× 1 ml of buffer B (0.5% TFA in 80% LC grade ACN and 20% LC grade water). Cartridges were equilibrated with 4× 1 ml of buffer A (0.5% TFA in LC grade water). Peptide samples were loaded on each cartridge. Cartridges were washed with 4× 1 ml of buffer A. Cartridges were removed from the vacuum manifold, placed in a 15-ml conical tube, and eluted by adding 1 ml of buffer B to the cartridge and spinning in a swing bucket rotor (Eppendorf 5910 R) at 300 rpm for 5 min. Eluent was transferred from the conical vial to a fresh 1.5-ml microfuge tube and dried using a vacuum centrifuge (Eppendorf Vacufuge Plus). Dried peptides were stored at −80°C until ready for analysis.

#### MS data acquisition

Data acquisition was the same as reported in the “MS data acquisition” section under the “Aging/cognition structural proteomics experiment” section, except that peptides were resuspended in 0.1% formic acid (Optima, Thermo Fisher Scientific) in LC grade water (Optima, Thermo Fisher Scientific) to an final concentration of 0.5 μg/μl (instead of 1 μg/μl), so a higher injection volume was used to still inject ~1 μg of peptides.

#### Data analysis: LFQ and nonrefoldability determination

For analysis of the data, FragPipe (version 22.0) was used. Two LFQ-MBR analyses were run—one with all the no-PK (trypsin-only) control samples and one with the LiP samples (which contain PK). Settings were the same as for the cognition experiment, as reported in the “Data analysis: LFQ and CASC determination” section, with the following exceptions. Samples were given the experiment label of “native_LiP,” “refold_1_min_LiP,” “refold_5_min_LiP,” and “refold_2hr_LiP,” depending on their refolding time, for the LiP LFQ (and similar labels for the control that indicated them as such).

For analysis of individual samples, the peptide.tsv and protein.tsv files from each individual sample were analyzed to gather quality metrics such as number of peptides, number of proteins, and percent half-trypticity, as described in the “Data analysis: LFQ and CASC determination” section. For analysis of the LFQs, we used the unmodified original version of the tool FLiPPR (*34*). Modifications, which were used for the cognition dataset, were unnecessary in this case since the same number of replicates was used for both native and refolded conditions, and the smaller number of replicates (*3*) is appropriate. Details regarding FLiPPR’s algorithm can be found in Manriquez-Sandoval *et al.* (*34*). FLiPPR treats features as described in the “Data analysis: LFQ and CASC determination” section, with the following changes. Missing data are not included in averages. Data imputation occurs only if all replicates are missing under one condition and all are present under the condition to which it was compared. In this case, the missing values are imputed with a random number from a Gaussian distribution centered at 10,000 with an SD of 1000.

Features in the combined ion file are merged to peptides by taking the median of the ratios of AU/AI ions, and *P* values are combined using Fisher’s method if the ratios agree in direction. Otherwise, the *P* value is set to one. Normalization of the abundance of a peptide from the LiP experiment occurs if the corresponding peptide in the control experiment has a fold change greater than twofold and a *P* value (using Welch’s correction for unequal population variances) less than 0.01.

FLiPPR outputs a summary file with a list of proteins, the number of valid peptides, and the number of significant peptides. A peptide is considered significantly different if the abundance change is greater than twofold between AU and AI (reported in log_2_) and if the adjusted *P* value (using Welch’s correction for unequal population variances and the Benjamini-Hochberg method for multiple hypothesis correction) is less than 0.05 (reported as −log_10_). The Benjamini-Hochberg correction is applied on a per-protein basis. If a protein has two or more significantly different peptides, then it is considered nonrefoldable, meaning that there is likely a structural difference between the refolded protein and the native protein. If a protein has two or more peptides detected but one or zero significantly different peptides, then it is considered refoldable, meaning that the native and refolded proteins likely have the same structure. Proteins with only one peptide matched are not considered to have enough information to be categorized as either refoldable or nonrefoldable.

### PTM analysis

#### Researches of data for PTMs

To determine whether PTMs confounded the LiP data analysis, we first re-searched all control (no PK added) samples for each region to look for PTMs, specifically phosphorylation and oxidation modifications. FragPipe (version 22.0) was used with the same settings as in the “Data analysis: LFQ and CASC determination” section, with the following modifications. We searched for phosphorylation and oxidation modifications in separate searches. For the phosphorylation analysis, we searched for dynamic phosphorylation modifications (+79.966 Da) on Ser, Thr, and Tyr residues. We also allowed methionine oxidation (+15.995 Da) and N-terminal acetylation (+42.011) as a dynamic modification. For the oxidation analysis, we allowed for dynamic oxidation modifications (+15.995 Da) on Met, Trp, Phe, and Tyr. We also allowed for N-terminal acetylation (+42.011) as a dynamic modification. For both searches, cysteine carbidomethylation was set as a static modification. Percent phosphorylated and percent oxidized peptides were determined by dividing the number of peptides with one (or more) of the modification by the total number of peptides detected in the peptide.tsv file generated by FragPipe for each sample in the LFQ.

#### Proximity analysis

The number of CASC and non-CASC sites near a modification (either phosphorylation or oxidation, regarded separately) was determined using an in-house Python script. All sites from the CASC study were considered, meaning all cut sites from PK and all centers of fully tryptic peptides. Peptides are considered significantly different (CASC) if the normalized peptide ratio effect size is two or greater between AU and AI, and the adjusted *P* value is less than 0.05. Otherwise, they are considered non-CASC sites. Next, for each significant site from the CASC study, we asked whether it was found within 15 amino acids on either side of a modification from the re-searched data. We repeated this with all nonsignificant sites from the CASC study. This was performed separately for each of the three hippocampal regions.

### Bioinformatics

#### Collection of protein metadata

Molecular weight, isoelectric point, instability index, and hydrophobicity of proteins were calculated using Biopython (*92*). Percent disorder was calculated by Metapredict (*40*). Domains for each protein were assigned using DomainMapper (*93*), which uses domain definitions from Evolutionary Classifications of Domains (ECOD) (*94*). Amyloidogenicity was assessed via PLAAC, a tool that looks for prion-like amino acid composition (*78*). Comparisons to turnover used protein turnover data from Kluever *et al.* (*84*).

#### GO analyses

All GO analyses were performed using ShinyGO version 0.82 (*72*). The species was set to rat [*R. norvegicus*, assembly name “Rat genes mRatBN7.2,” taxonomy ID 10116, sourced from ENSEMBL (*95*)]. Settings for all searches were an FDR cutoff of 0.05, a minimum pathway size of 2, a maximum pathway size of 2000, and redundancy removal. For the analysis, a list of accession numbers for proteins with more than one significant peptide representing nonrefoldable proteins (from the 5-min refoldability native to refolded comparison) was supplied to ShinyGO, along with a reference set of the accession numbers of all proteins from the appropriate dataset with greater than one peptide detected. We examined cellular component GO terms, in addition to KEGG pathways (*73*) and RGD terms (*74*).

#### CASC/refoldability overlap and proximity analysis

CA1 CASC and 5-min refoldability overlap were determined by considering all proteins that are considered CASC (or nonrefoldable for the refoldability study). A protein is considered CASC or nonrefoldable if there is more than one peptide for that protein where the normalized peptide ratio effect size is two or greater (between AU and AI for CASC and between 5-min refolded and native for refoldability), and the adjusted *P* value is less than 0.05. Other proteins are considered non-CASC (or refoldable) if they have greater than one peptide, but one or fewer are significantly different. The overlap was considered to be proteins in the intersection of sets of the appropriate category (for example, the intersection of the set of CA1 CASC proteins and the set of 5-min nonrefoldable proteins). Proteins that were only identified in either the CA1 CASC study or the 5-min refoldability study, but not in the other, were not considered in this analysis.

For the CASC and refoldability proximity analysis, we considered sites from the CA1 CASC study and the 5-min refoldability study. We considered sites from the CA1 CASC study to be the location of the PK cut site of the significant peptide or the center of the significant peptide if it is fully tryptic. For each significant site, we asked whether the site is located within five amino acids on either side of a nonrefoldable site, then whether it is within five amino acids on either side of a refoldable site, whether it is located within five amino acids on either side of both a nonrefoldable site and a refoldable site, or whether it is not located near either. We then asked this for nonsignificant sites as well. Nonrefoldable and refoldable sites were considered to be significant and nonsignificant peptides, respectively, from the 5-min refoldability study.

#### Structural visualization

Protein structures were generated from AlphaFold version 2 (*41*) from their accession numbers. Accession numbers used were Q9QX69 (glutathione *S*-transferase LANCL1), P61078 (ubiquitin-conjugating enzyme E2 D3), P07895 (SOD2), P30835 (Pfkl), and P63081 (Atp6v0c). Structures were visualized using PyMOL version 1.8.3.2. Significant sites from the CA1 CASC study (PK cut sites of significant peptides or centers of fully tryptic significant peptides) were represented as light-blue spheres on the structure. Significant sites from the 5-min refoldability study (nonrefoldable sites) were represented as black spheres on the structure. Sites that were significant in both the CA1 CASC study and 5-min refoldability study were represented as dark-blue spheres.
