# Supplementary material for: Proteins with cognition-associated structural changes in a rat model of aging exhibit reduced refolding capacity
Source: Sci Adv. 2025 Jul 11;11(28):eadt3778. doi: 10.1126/sciadv.adt3778 (PMC12248298; doi:10.1126/sciadv.adt3778)
Supplement: Supplementary file 1 — Supplementary Methods Figs. S1 to S9 Tables S1 to S3 Legends for data S1 to S4 [file sciadv.adt3778_sm.pdf]

Science Advances
AAAS

Supplementary Materials for

Proteins with cognition-associated structural changes in a rat model of aging exhibit reduced refolding capacity

Haley E. Tarbox *et al.*

Corresponding author: Stephen D. Fried, sdfried@jhu.edu

Sci. Adv. **11**, eadt3778 (2025)
DOI: 10.1126/sciadv.adt3778

The PDF file includes:

Supplementary Methods
Figs. S1 to S9
Tables S1 to S3
Legends for data S1 to S4

Other Supplementary Material for this manuscript includes the following:

Data S1 to S4

Supplementary Methods

S1. Aggregation Assay

S1a. Preparation of Native and Refolded Hippocampal Extracts

Frozen whole hippocampi from young (approx. 6 month old) rats were removed from the -80°C freezer and immediately added to 750µL of chilled lysis buffer (20mM Tris pH 8, 100mM NaCl, 2mM $MgCl_2$) containing protease inhibitors (PMSF 0.5mM final concentration (f.c.), bestatin 0.05mM f.c., E64 0.015mM f.c., all diluted from 100X stock in DMSO) and DNase (0.1 mg/mL f.c., diluted from 100X stock in MPW) in a 1mL dounce homogenizer (Wheaton USA) on ice. Samples were vigorously homogenized on ice. Homogenized tissue was moved to a 1.5mL microfuge tube and clarified at 16000x*g* for 15 min at 4°C to remove insoluble cellular debris. The supernatant was moved to a fresh 1.5mL microfuge tube and kept on ice. Protein concentrations of the samples were determined using the bicinchoninic acid (BCA) assay, according to the manufacturer's protocol (Pierce Rapid Gold Protein Assay Kit, Thermo Scientific A53225) and using bovine serum albumin (BSA) as a standard. Lysates were normalized to 2 mg/mL by dilution with lysis buffer.

To prepare native samples, normalized lysate was diluted to 0.115 mg/mL f.c. using native dilution buffer (20mM Tris pH 8, 100mM NaCl, 2mM $MgCl_2$, 1.061mM DTT, 0.0637M guanidinium chloride (GdmCl)). This buffer has a small amount of GdmCl to match the concentration of GdmCl that will be present after refolding of unfolded samples. Final concentrations of all components were 0.115 mg/mL protein, 20mM Tris pH 8, 100mM NaCl, 2mM $MgCl_2$, 0.06mM GdmCl, and 1mM DTT. Native samples were incubated at room temp overnight.

To prepare refolding samples, proteins were first unfolded. Initially, 500µg of protein was added to a fresh 1.5mL microfuge tube, and 25mg of GdmCl powder and 0.61µL of a 700mM DTT stock was added. Refolding samples were placed in a Vacufuge Plus (Eppendorf) and volume was reduced to 43µL. Final concentrations of all components were 11.5 mg/mL protein, 115mM Tris pH 8, 575mM NaCl, 11.5mM $MgCl_2$, 6M GdmCl, and 10mM DTT. Samples were incubated at room temp overnight to ensure complete unfolding of the samples.
To refold the samples, samples were diluted 100X in refolding dilution buffer (19.03mM Tris pH 8, 95.14mM NaCl, 1.9mM $MgCl_2$, 0.909mM DTT) by adding 10µL of unfolded sample into 990µL of refolding dilution buffer. Final concentrations of all components were 0.115 mg/mL protein, 20mM Tris pH 8, 100mM NaCl, 2mM $MgCl_2$, 0.06mM GdmCl, and 1mM DTT. All concentrations are the same as the native samples. Refolded samples were incubated for two hours at room temp to allow proteins to refold.

S1b. Determination of Aggregation Percentage

Native and refolded samples were centrifuged at 16000x*g* for 15 min at 4°C to collect any aggregated material. The supernatant was removed, taking care not to disturb the pellet containing aggregated proteins. The pellet was washed with 200µL lysis buffer, which reduces the potential interference from reducing agents in the sample with the BCA assay. The pellet was resuspended in 75µL of 8M urea in MPW. Protein concentrations were determined by the BCA assay, as previously described. There were three biological replicates for the native and refolded

samples, and the BCA was performed with technical triplicates. The percent aggregation was determined by determining the total amount of protein in the pellet and dividing by the total amount of protein in the initial native or refolded sample. This calculation yields the percent protein that was precipitated in the sample. Data are reported as mean ± standard deviation. T-tests were performed in Prism 10 (Graphpad) using a parametric unpaired T-test with Welch's correction for unequal population variances (the same standard deviation is not assumed).

S2. Reproducibility Experiment

Three AU hippocampi with subfield dissections were obtained in order to determine biological variation (between the three samples) and technical variation (of LiP, performed multiple times on the same lysate, and of the instrument, by shooting identical samples more than once). Each CA1 region was lysed by Dounce homogenization vigorously on ice in 0.5mL chilled lysis buffer (20mM Tris pH 8.0, 100mM NaCl, 2mM $MgCl_2$) in the presence of protease inhibitors (0.5mM PMSF, 0.015mM E64, 0.05mM bestatin f.c.) and 0.1 mg/mL f.c. DNase. Lysate was clarified for 15 min at 15000xg and 4°C. remove insoluble cellular debris. The supernatant was moved to a fresh 1.5mL microfuge tube and allowed to sit at room temp for at least two hours and twenty minutes before limited proteolysis (LiP), to ensure that PMSF had sufficient time to hydrolyze (approx. four half lives) and would not inhibit our protease during the LiP step. During this time, protein concentrations of the samples were determined using the bicinchoninic acid (BCA) assay, according to the manufacturer's protocol (Pierce Rapid Gold Protein Assay Kit, Thermo Scientific A53225). Supernatant was normalized to 0.75 mg/mL with lysis buffer. Each sample was split into six 100µL aliquots, three for non-LiP (no PK) and three for LiP. After adequate time for hydrolysis of PMSF has passed (so that PMSF will not inhibit PK, at least 2 h 20 min (approx. 4 half lives at pH 8.0)), LiP can be performed. Proteinase K (PK, Thermo Scientific 17916, previously prepared as 1 mg/mL stock in 1:1 v/v of lysis buffer to 20% glycerol, aliquoted, flash frozen and stored at -20°C) was diluted to 0.25 mg/mL such that 3uL of the dilution would contain 1:100 w/w of PK to the amount of sample to be digested (in this case, either 0.75µg). For each of the three LiP reactions per sample, 3µL of 0.25 mg/mL PK was placed at the bottom of a fresh 1.5mL microfuge tube. 100µL of normalized sample was added to the tube containing PK and pipetted rapidly up and down seven times to mix thoroughly. This mixture was incubated for exactly one minute before the tube was added to a 105°C mineral oil bath for five minutes to quench the PK reaction. For each sample, three corresponding no-PK, non-LiP controls were performed. In this case, 100µL of normalized lysate was added to a fresh 1.5mL tube without PK and then added to the oil bath for 5 minutes. After 5 minutes, all samples were removed from the oil bath and quickly centrifuged to collect condensation and transferred to a new 2mL tube containing 76mg urea (8M urea f.c., 314µL f.v.). To each sample in 8M urea, in order to reduce disulfides, dithiothreitol (DTT) was added to 10mM f.c. (2.25µL added from a freshly made 700mM DTT stock) and incubated in a thermomixer for 30 min at 37°C and 700 rpm. To cap cysteines, iodoacetamide (IAA) was added to a f.c. of 40mM (9µL added from a freshly made 700mM IAA stock) and incubated at room temperature (rt) in the dark for 45 min. Samples were diluted to 2M urea f.c. by adding 505µL of a freshly-made 100mM ammonium bicarbonate (ambic) stock. To each sample, 1.5µg trypsin (New England Biolabs) was added (1:50 w/w of trypsin to protein). Samples were incubated overnight (approximately 16 hours) in a thermomixer at 25°C and 700 rpm. Digested samples were acidified with trifluoroacetic acid (TFA, Acros) to a 1% f.c. by volume by adding 6.6µL TFA. Peptides were desalted using Sep-

Pak Vac 1cc (50mg) C18 cartridges (Waters) and dried down and stored at -80°C, as described in section 3b of the main text methods.

Samples were resuspended in 75µL 0.1% formic acid and data was acquired as described in section 3c, except that samples were shot in technical duplicate for one of the three biological replicates.

Eleven label-free quantification (LFQ) analyses were performed, one with all the no-PK (trypsin only) controls for the biological replicate analysis, one with the LiP samples (which contain PK) for the biological replicate analysis, six for each of the technical replicates (each compares the same sample that was shot twice on the instrument), and three for LiP reproducibility (one for each animal that compares the LiP performed on samples from the same animal). FragPipe (version 22.0) was used. Default settings were used except where specified otherwise. Settings were the same as in section 3d of the main text methods.

For the instrument variation (technical) analyses, the same sample that was injected twice was compared. Abundance ratios were generated by dividing the ion intensity for each sample from the combined_peptide.tsv file generated by FragPipe. For the LiP variation analyses, LiP replicates from the same sample were compared. Abundance ratios were compared by dividing the MaxLFQ intensity for the desired ratio. In the case of missing data, values of 0 were replaced by 1000 (this prevents division by 0 errors). Ratios are reported in log2.

For the biological replicate analysis, one of the LFQs, the control LFQ, compared the three non-PK, trypsin-only, samples for all the biological replicates. The LiP LFQ compared the three LiP samples, where PK was added. We ran FLiPPR (*34*) , inputting the FragPipe outputs, to generate abundance ratios and adjusted P-values for peptides (details can be found in section 3d of the main text methods). Missing data are not considered for calculating abundance ratios. Data imputation occurs only if all replicates are missing in one animal and all are present in the animal to which it was compared. In this case, the missing values are imputed with a random number from a Gaussian distribution centered at 10,000 with a standard deviation of 1,000. Features in the combined ion file are merged to peptides by taking the median of the ratios of ions, and P-values are combined using Fisher's method if the ratios agree in direction. Otherwise, the P-value is set to one. Normalization of the abundance of a peptide from the LiP experiment occurs if the corresponding peptide in the control experiment has a fold-change of greater than 2-fold and the p-value (using Welch's correction for unequal population variances) is less than 0.01. FLiPPR generates a peptide file where the effect size (as a ratio of averages, reported in log2) and adjusted p-values (reported as -log10, assessed using t tests with Welch's correction for unequal population variances and the Benjamini-Hochberg method for false discovery correction) are reported.

Supplemental Figures

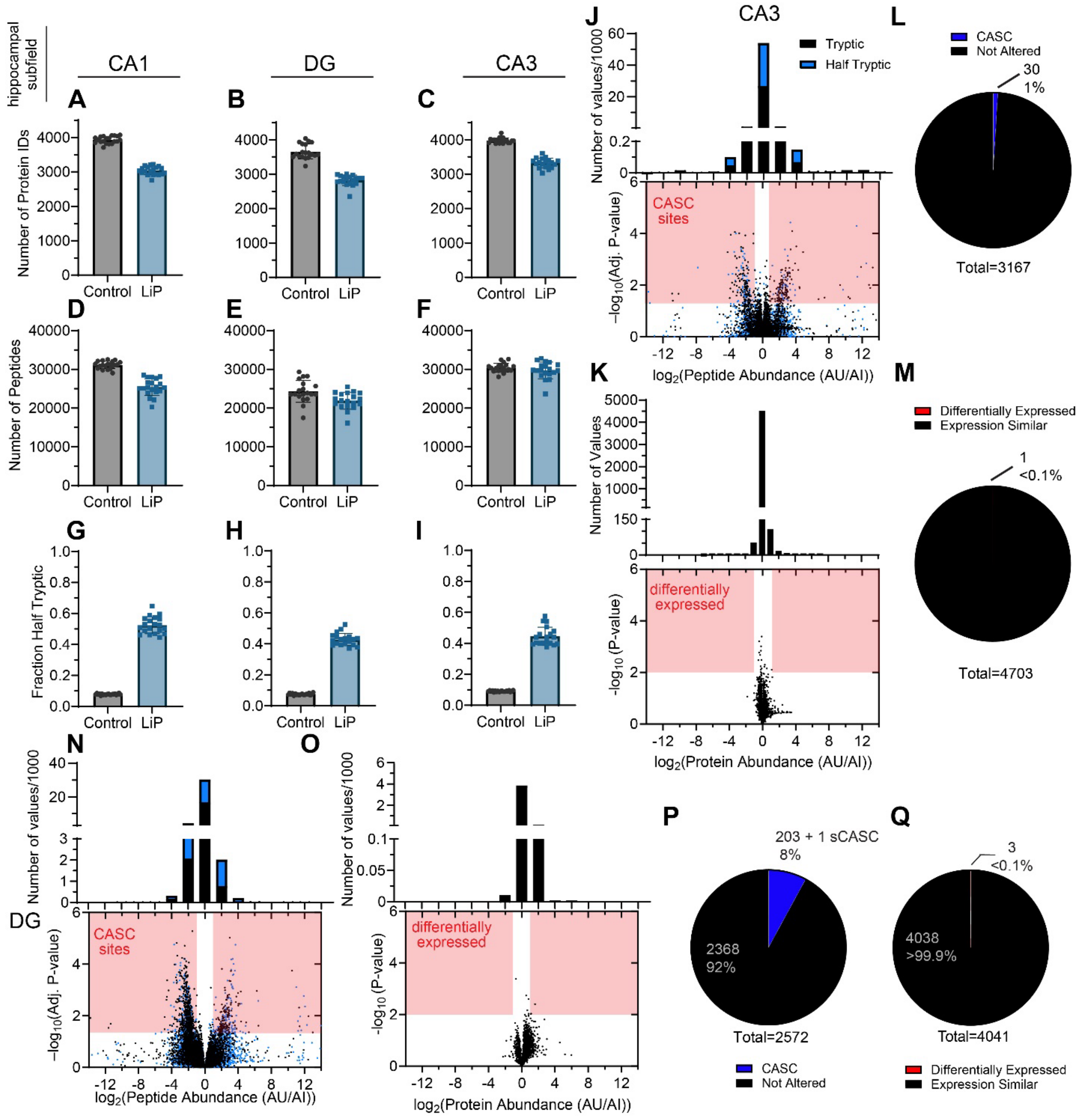

Fig. S1. Quality Controls for Limited Proteolysis Mass Spectrometric (LiP-MS) Study of the Aging Rat's Hippocampal Proteome. (A-C) Number of proteins identified in individual mass spec runs on three hippocampal subfields ((A) CA1; (B) DG; (C) CA3) filtered to an FDR of 5% by ProteinProphet. Each point represents an individual subject (biological replicate). Bars represent average, errors bars represent standard deviations. (D-F) Number of peptides identified in individual mass spec runs on three hippocampal subfields ((D) CA1; (E) DG; (F) CA3) filtered to an FDR of 5% by PeptideProphet. Each point represents an individual subject (biological replicate). Bars represent average, errors bars represent standard deviations. (G-I) Of

the peptides identified in an individual mass spec run on three hippocampal subfields ((G) CA1; (H) DG; (I) CA3), the fraction that are half-tryptic (that is, one cut-site is non-tryptic and presumed to arise from Proteinase K). Bars represent average and error bars represent standard deviations. In panels (A)-(I), “Control” denotes samples processed without limited proteolysis (only digested with trypsin) and “LiP” denotes samples subjected to limited proteolysis with Proteinase K, then trypsin digest. (J) Volcano plot showing changes in peptide abundance of tryptic (black) and half-tryptic (blue) peptides in the CA3 subfield between AU and AI rats. Dots that fall in the regions in red are deemed significant based on effect size (>2-fold) and adjusted $p < 0.05$ by t-test with Welch’s correction for unequal population variance and false discovery rate (FDR) corrected by the Benjamini-Hochberg (BH) procedure. (K) Volcano plot showing changes in protein abundance in the CA3 subfield between AU and AI rats. Dots that fall in the regions in red are deemed significant based on effect size (>2-fold) and $p < 0.01$ by t-test with Welch’s correction for unequal population variance. (L) Number of proteins with cognition-associated structural changes (CASC) in the CA3 subfield. CASC proteins have two or more peptides with significant changes. All proteins must have two or more peptides detected. (M) Number of proteins with different measured abundance in AU vs AI subjects in the CA3 subfield. (N) Volcano plot showing changes in peptide abundance of tryptic (black) and half-tryptic (blue) peptides in the DG subfield between AU and AI rats. Dots that fall in the regions in red are deemed significant based on effect size (>2-fold) and adjusted $p < 0.05$ by t-test with Welch’s correction for unequal population variance and false discovery rate (FDR) corrected by the Benjamini-Hochberg (BH) procedure. (O) Volcano plot showing changes in protein abundance in the DG subfield between AU and AI rats. Dots that fall in the regions in red are deemed significant based on effect size (>2-fold) and $p < 0.01$ by t-test with Welch’s correction for unequal population variance. (P) Number of proteins with cognition-associated structural changes (CASC) in the DG subfield. CASC proteins have two or more peptides with significant changes. All proteins must have two or more peptides detected. (Q) Number of proteins with different measured abundance in AU vs AI subjects in the DG subfield.

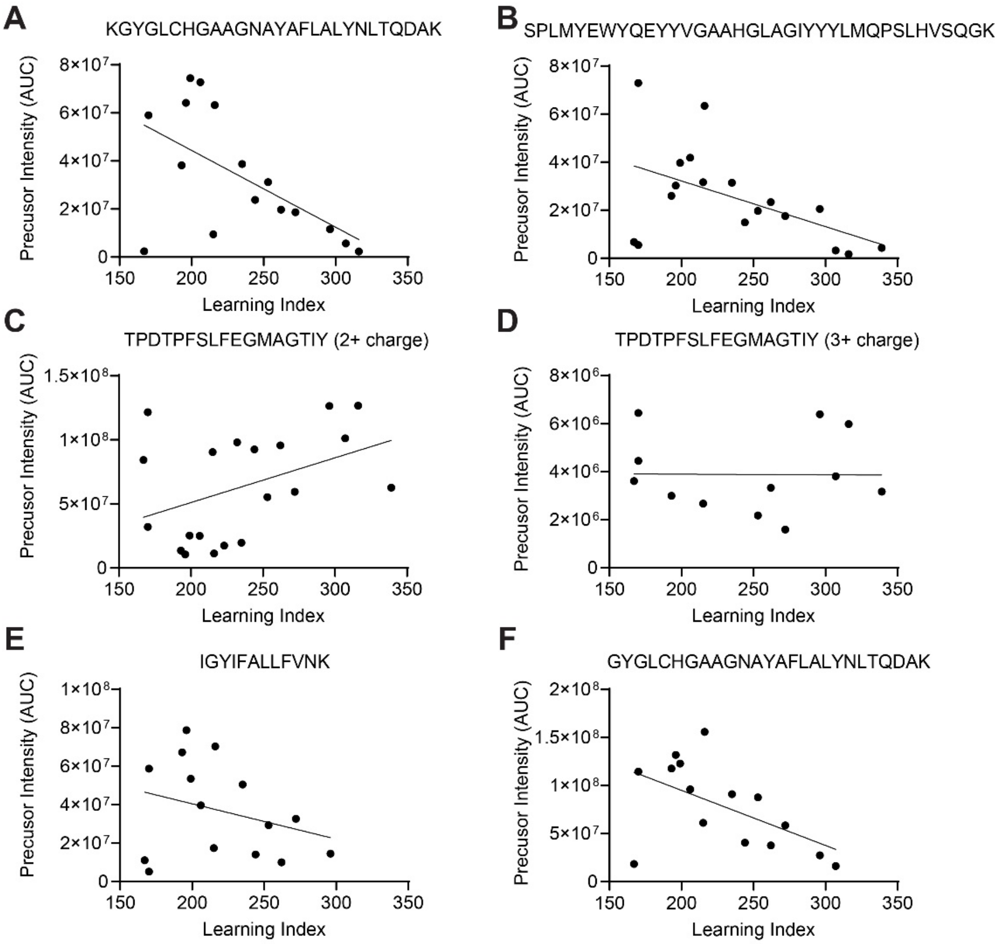

G

LANCL1 Peptide	p (Categorical, Welch's t-test)	p (LSRL)	p (LSRL, excluding missing)	Number Missing Values
KGYGLCHGAAGNAYAFLALYNLTQDAK	0.00034	0.052	0.019	4
SPLMYEWYQEYYVGAAHGLAGIYYYLMQPSLHVSQGK	0.0053	0.048	0.032	2
TPDTPFSLFEGMAGTIY (2+ charge state)	0.0035	0.064	0.064	0
TPDTPFSLFEGMAGTIY (3+ charge state)		0.33	0.98	8
IGYIFALLFVNK	0.0052	0.025	0.29	5
GYGLCHGAAGNAYAFLALYNLTQDAK	0.0048	0.041	0.034	5

Fig. S2. Use of Regression to Assess a Peptide's CASC status. (A-F) For each peptide identified to be significantly different in Glutathione S-transferase LANCL1 (Uniprot accession Q9QX69), precursor intensity (Area Under Curve, AUC) of that peptide is plotted versus learning index from the Morris water maze test for each rat. A least squares regression line (LSRL) is fit for each peptide. Missing values are omitted. (G) Table shows for each peptide: the unadjusted p-value using Welch's t-test from the categorical analysis (comparing AU versus AI), the LSRL p-values both with and without missing values from the continuous analysis using learning indexes, and the number of missing values for each peptide.

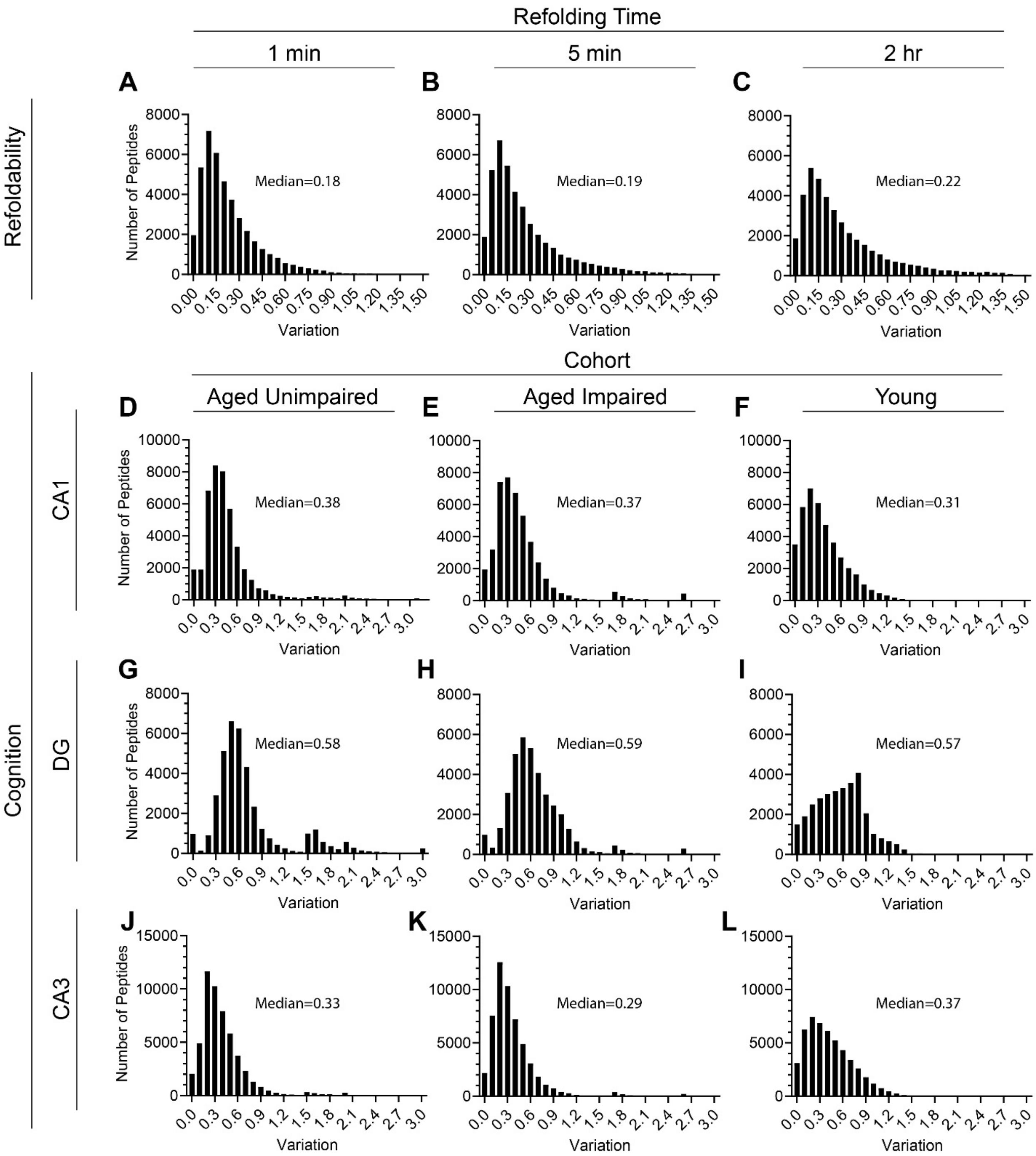

Fig. S3. Analysis of Variation. Histograms show the coefficients of variation (standard deviation divided by mean) for peptide abundance in limited proteolysis samples across the replicates for all conditions surveyed in this study. Median coefficient of variation reported. (A-C) Coefficients of variation across 3 replicates of global refolding reactions, diluted from denaturant and allowed to refold for (A) 1 min; (B) 5 min; (C) 2 h. (D-L) Coefficients of variation across biological replicates from 3 hippocampal subfields (CA1, panels (D-F); DG, panels (G-I); CA3, panels (J-L)) collected from rats from 3 cohorts (aged unimpaired, panel (D), 10 replicates, panels (G),(J), 9 replicates; aged impaired, panels (E),(H),(K), 7 replicates; young, panels (F),(I),(L), 3 replicates).

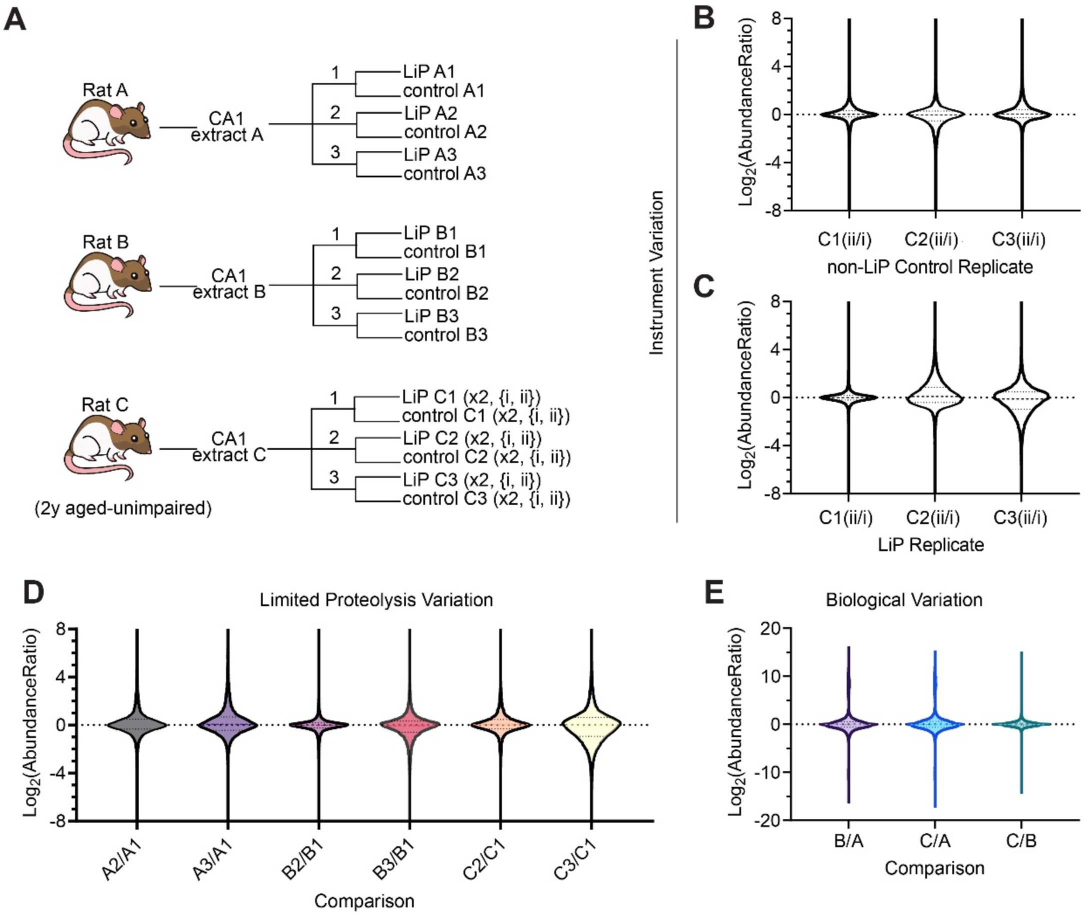

Fig. S4. Reproducibility of Limited Proteolysis Methodology. (A) Scheme illustrating experimental design of the reproducibility study. Three biological replicates (all cognitively unimpaired rats, aged 2 y) were sacrificed, and the CA1 subfield of their hippocampi extracted. These biological replicates were subjected to 3 technical replicates of preparing LiP samples and "control" (non-LiP trypsin-only) samples. For rat C, these 3 technical replicates were also subjected to analytical duplicates (injected on the LC-MS/MS twice). (B, C) Analytical variability in peptide abundance between two injections of non-LiP control samples, (B); and between two injections of LiP samples, (C). (D) Technical variability in peptide abundance following replicate LiP reactions derived from the same biological replicate. (E) Biological variability in peptide abundance across different subjects' CA1 hippocampal region. Each subject consists of three technical replicates for both LiP and control.

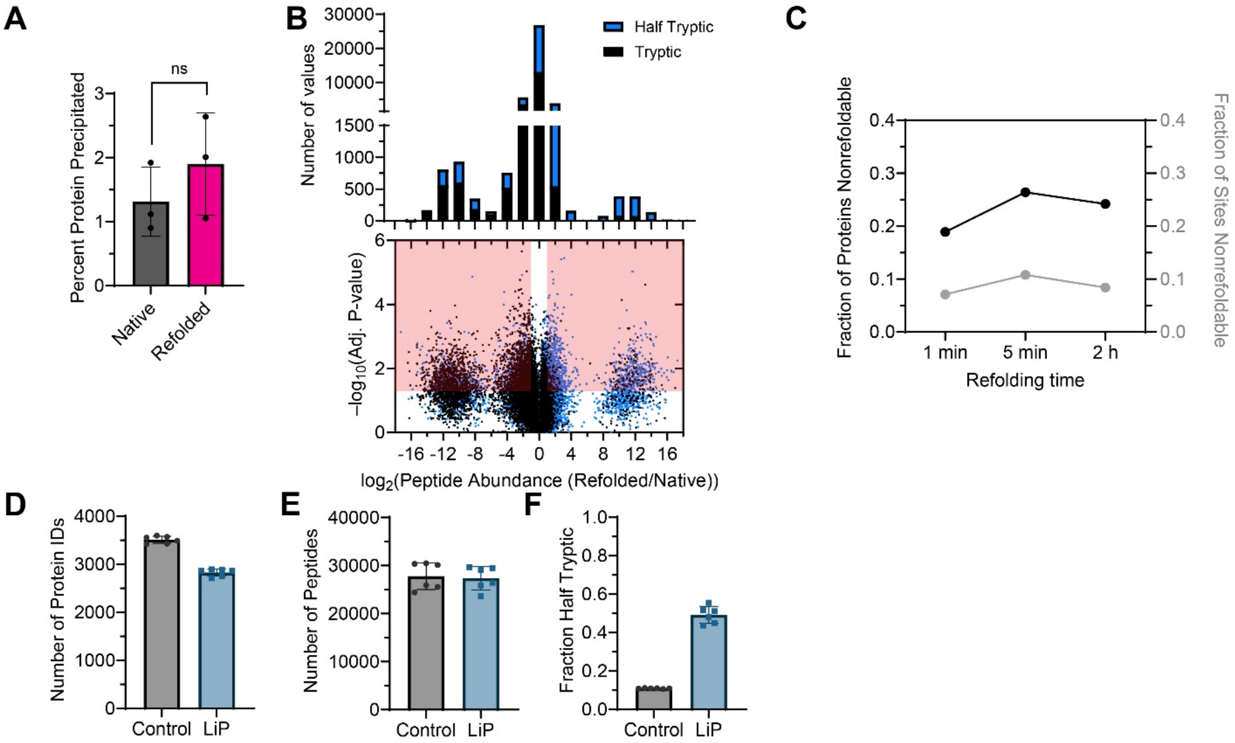

Fig. S5. Quality Controls for Limited Proteolysis Mass Spectrometric (LiP-MS) Refolding Experiment on Rat Hippocampal Proteome. (A) Percent of protein content that precipitates from clarified rat hippocampal extract either before (gray) or after (pink) a global refolding cycle conducted by incubating in 6 M guanidinium chloride followed by 100-fold dilution (see *Methods* Section 4 for details). Aggregated protein was collected by centrifugation, resuspended in 8 M urea, and quantified by bicinchoninic acid (BCA) assay. Dots represent biological replicates, bars represent averages, and error bars represent standard deviations. (B) Volcano plot showing changes in peptide abundance of tryptic (black) and half-tryptic (blue) peptides in the rat hippocampus following a global unfolding-refolding cycle relative to a native reference using a 5 min refolding time. Dots that fall in the regions in red are deemed significant based on effect size (>2-fold) and adjusted $p < 0.05$ by t-test with Welch's correction for unequal population variance and false discovery rate (FDR) corrected by the Benjamini-Hochberg (BH) procedure. Histograms show number of peptides possessing abundance ratios in the various ranges. (C) Fraction of proteins nonrefoldable (black, using the criterion that the protein has two or more peptides with significant changes in proteolytic susceptibility in the refolding reactions) and fraction of peptides/sites nonrefoldable (gray, using the criterion the peptide has >2-fold change in abundance (adjusted $p < 0.05$ by t-test with Welch's correction and false discovery rate (FDR) corrected by the Benjamini-Hochberg (BH) procedure) in the refolding reactions) as a function of refolding time. Each time-point used three separate refolding reactions performed on biological triplicate (i.e., separate hippocampi from separate rats). (D) Number of proteins identified in individual mass spec runs used for *in vitro* refolding experiments filtered to an FDR of 5% by ProteinProphet. Bars represent average, errors bars represent standard deviations. (E) Number of peptides identified in individual mass spec used for *in vitro* refolding experiments filtered to an FDR of 5% by PeptideProphet. Bars represent average, errors bars represent standard deviations. (F) Of the peptides identified in an individual mass spec run used for *in vitro* refolding experiments, the fraction that are half-tryptic (that is, one cut-site is non-tryptic and

presumed to arise from Proteinase K). Bars represent average, errors bars represent standard deviations. In panels (D)-(F), “Control” denotes samples processed without limited proteolysis (only digested with trypsin) and “LiP” denotes samples subjected to limited proteolysis with Proteinase K, then trypsin digest.

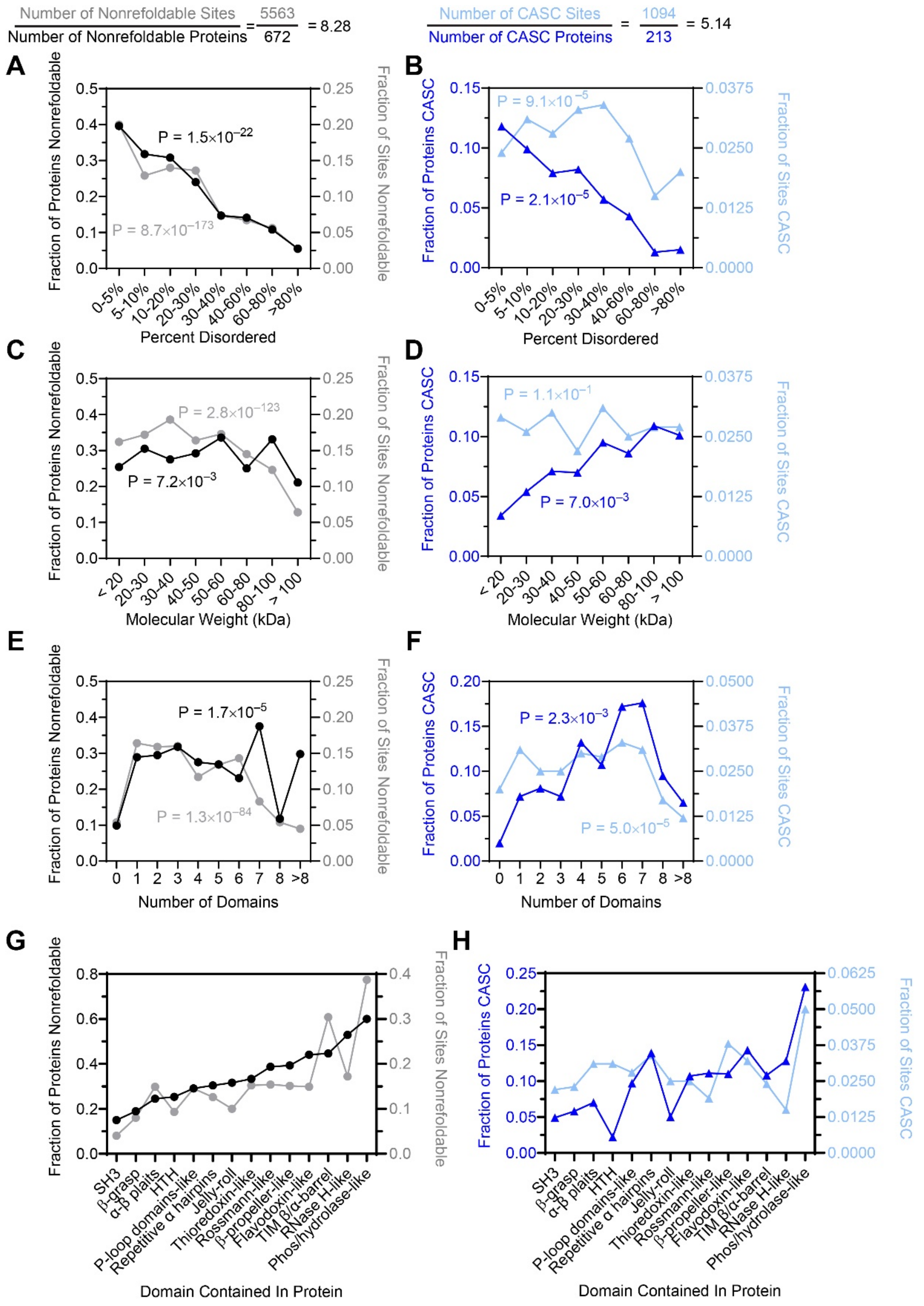

Fig. S6. Robustness Analysis of Trends Found on Nonrefoldable Proteins and Cognition-Associated Structurally Changed (CASC) Proteins. (A,C,E,G) Plots provide the fraction of proteins within a given category that are nonrefoldable after allowed 5 min to refold from denaturant (black, left y-axis) and the proportion of sites (peptides) within a given category that

had significantly altered proteolytic susceptibility after 5 min of refolding (gray, right y-axis). Proteins and peptides are divided as a function of: (A) percent disorder according to Metapredict (*40*), (C) molecular weight, and (E) number of domains, based on ECOD (*94*) using DomainMapper (*93*). P-values (according to chi-square test) against the null hypothesis that nonrefoldability is independent of the categorical variable in question are provided. (G) Proteins (or peptides) assessed based on whether they contain a domain of a given topology (or come from a protein that contains a domain of a given topology). These categories are not mutually exclusive since some proteins contain multiple domains. (B,D,F,H) Plots provide the fraction of proteins within a given category that are CASC in the CA1 region (blue, left y-axis) and the proportion of sites (peptides) within a given category that had significantly altered proteolytic susceptibility in aged impaired rats compared to aged unimpaired (light blue, right y-axis). Proteins and peptides are divided as a function of: (B) percent disorder according to Metapredict (*40*), (D) molecular weight, and (F) number of domains, based on ECOD (*94*) using DomainMapper (*93*). P-values (according to chi-square test) against the null hypothesis that CASC is independent of the categorical variable in question are provided. (H) Proteins (or peptides) assessed based on whether they contain a domain of a given topology (or come from a protein that contains a domain of a given topology). These categories are not mutually exclusive since some proteins contain multiple domains.

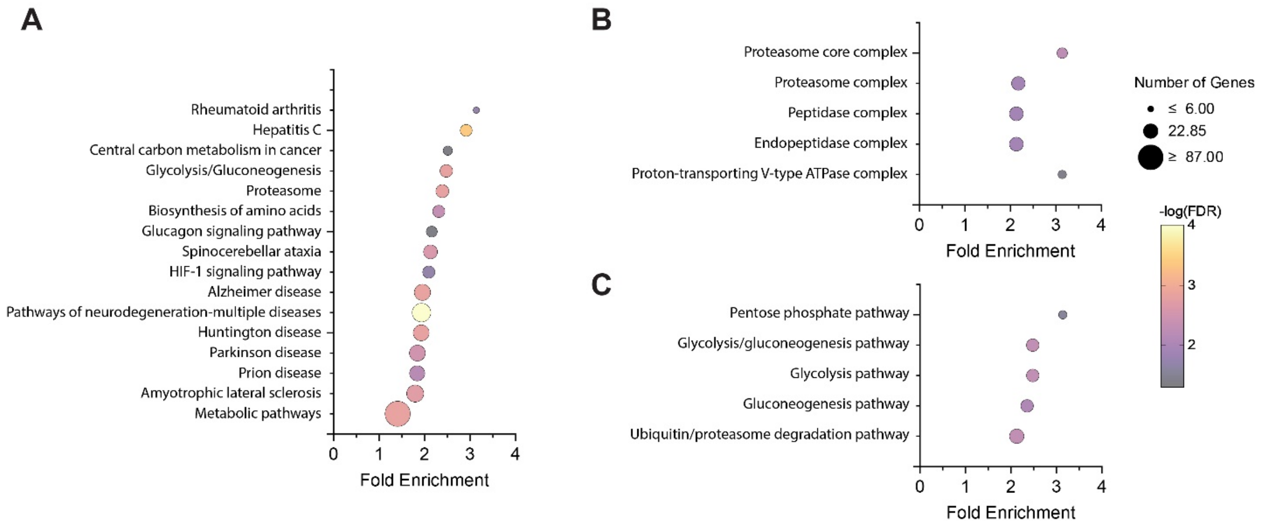

Fig. S7. Gene Ontology Analysis of Nonrefoldable Proteins. Analysis is based on the 672 nonrefoldable proteins identified in global refolding experiments on the rat hippocampal proteome, with a reference set composed of all 2429 proteins quantified with 2 or more peptides across the 6 samples used for the experiment (biological triplicates of native and refolded (5 min) extracts) using ShinyGO (*72*). (A) Using the Kyoto Encyclopedia of Genes and Genomes (KEGG) database (*73*). (B) Using the cellular component GO terms. (C) Using the Rat Genome Database (RGD) terms (*74*).

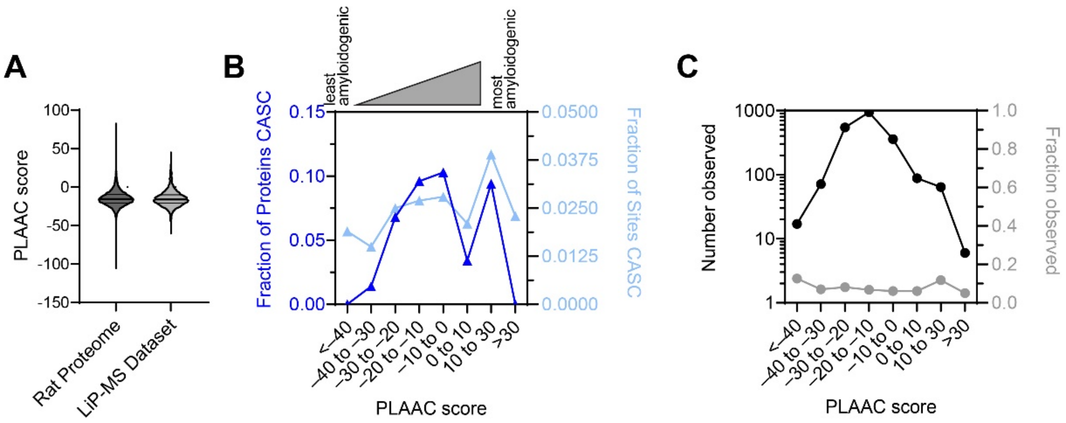

Fig. S8. Association between CASC proteins and amyloidogenicity. Amyloid propensity is based on the log-likelihood score of the PLAAC algorithm (*78*) using default parameters. (A) Distribution of PLAAC scores for all proteins in the rat proteome versus those represented in the LiP-MS aging study presented here. (B) Plot provides the fraction of proteins that are CASC in the CA1 region (blue, left y-axis) and the proportion of sites (peptides) that had significantly altered proteolytic susceptibility in aged impaired rats compared to aged unimpaired (light blue, right y-axis), categorized by each protein's PLAAC log-likelihood score. Proteins that are predicted to be most amyloidogenic (high positive PLAAC score) are no more likely to be CASC than less amyloidogenic proteins (negative PLAAC score). (C) Number of proteins observed in each of the PLAAC score bins (black) in the LiP-MS aging study, and the percentage of all rat proteins within a PLAAC score bin that are observed in the LiP-MS aging study (gray). This shows that although there are, for instance, many fewer proteins with PLAAC > 30, this category is nevertheless observed with the same frequency as other categories.

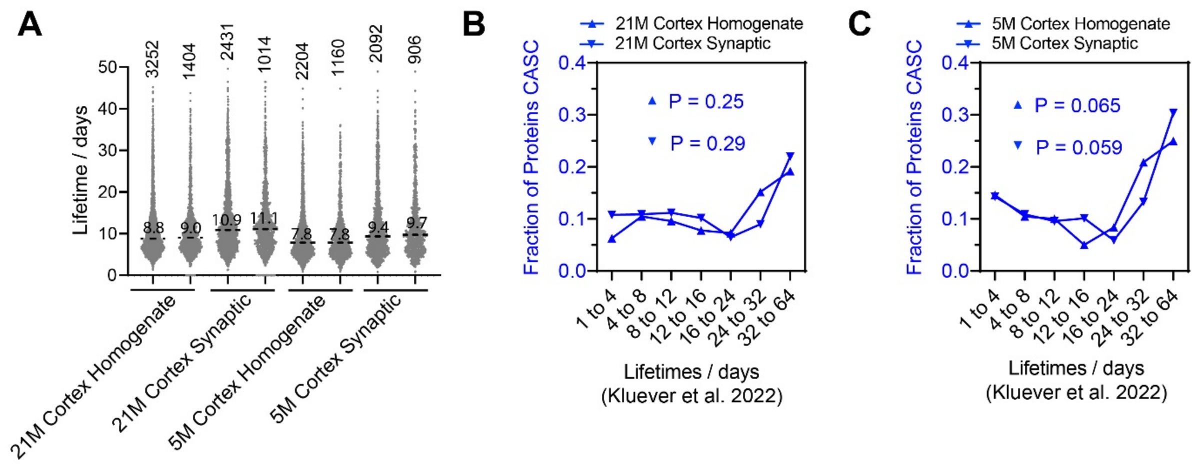

Fig. S9. Association between CASC proteins and lifetime. Lifetimes are based on the study by Kluever et al. (*84*), by referring to the values in "tHalf" columns in Table S1 and Table S3 from ref. (*84*). As the previous work used mice instead of rats, we used InParanoiDB 9 to identify the rat orthologue for each mouse protein. (A) Distribution of protein lifetimes observed by Kluever et al. (*84*) for proteins isolated from mouse cortex, either from animals aged to 21 months (old) or 5 months (young), or from total cortex homogenates or synaptic fractions. To the right of each, the distribution of protein lifetimes from that experiment that were also assessed in the LiP-MS aging study with more than one peptide identified. The median lifetime and the total number of proteins measured are noted for each distribution. (B) Plot provides the fraction of proteins that are CASC in the CA1 region (blue, left y-axis), categorized by the orthologous protein's lifetime in the mouse cortex. P-values (according to chi-square test) against the null hypothesis that CASC is independent of the lifetime category. (C) As panel B, except using lifetimes measured for rats aged to 5 months instead of 21 months.

Supplemental Tables

Accession	Gene Name	Protein Description
Q9QX69	Lancl1	Glutathione S-transferase LANCL1
P63081	Atp6v0c	V-type proton ATPase 16 kDa proteolipid subunit c
Q6AYS7	Acy1a	Aminoacylase-1A
P05545	Serpina3k	Serine protease inhibitor A3K
P07895	Sod2	Superoxide dismutase [Mn], mitochondrial
P30835	Pfkl	ATP-dependent 6-phosphofructokinase, liver type
P61078	Ube2d3	Ubiquitin-conjugating enzyme E2 D3
Q8R491	Ehd3	EH domain-containing protein 3
B0BNA5	Cotl1	Coactosin-like protein
D4A8Y6	Vcan	Versican core protein
A0A0G2JV77	Mink1	Non-specific serine/threonine protein kinase
A0A0G2JWA3	Xpo7	Exportin 7
A0A8I5Y830	Psma7	Proteasome subunit alpha type
A0A8I6A0T8	Serpinb9	Serpin family B member 9
A0A0H2UI36	AABR07060872.1	Ig-like domain-containing protein

Table S1. List of CA1 sCASCs. Table reports the uniprot accession numbers, gene names, and protein descriptions of proteins found to be sCASCs in the CA1 regions. sCASC proteins contain two or more significantly different peptides (greater than two-fold normalized abundance change in either direction, and adjusted p-value of less than 0.05 (using Welch's correction for unequal population variance and false discovery rate (FDR) correction by the Benjamini-Hochberg (BH) procedure)) between the AU and AI samples. sCASCs differ from the general CASCs in that the analysis does not impute any values for missing data.

Region	Analysis	Num. of (s)CASC	Random 1	Random 2	Random 3	Average Randoms	Ratio Random/True
CA1	Strict	15	1	0	0	0.33	0.02
CA1	General	213	60	50	17	42.33	0.20
DG	Strict	1	61	4	1	22	22
DG	General	204	1043	407	179	543	2.66
CA3	Strict	0	1	0	0	0.33	--
CA3	General	30	80	47	29	52	1.73

Table S2. True versus null analyses of (s)CASCs. Reports the number of proteins with two or more significant peptides in the "true" analysis, where all samples are correctly assigned labels of age unimpaired (AU) or age impaired (AI) as based on the Morris water maze test, or in the random analyses, where samples are randomly assigned AU or AI labels. In the AI category, 3 of 7 samples are indeed truly AI in Random1, 2 of 7 in Random2, and 3 of 7 in Random3. "Strict" refers to the analysis with no imputation for missing data, and hits in this dataset are sCASCs, whereas the "general" analysis does include missing value imputation in the workflow when conditions for imputation are met, and hits in this dataset are CASCs.

	Max Missing (AI, AU)	Imp. 1 (# missing)	Imp. 2 (# missing)	Imp. Type	Sig. FC	# CA1 CASC	# Random1 CASC	Ratio R1/True
1 sCASC	3, 5	None	None	N/A	2	15	1	0.067
2	4, 6	None	None	N/A	2	18	1	0.056
3	3, 5	None	None	N/A	1.5	29	4	0.14
4	5, 8	None	None	N/A	2	17	2	0.12
5	2, 3	None	None	N/A	2	12	0	0
6 CASC	3, 5	AI>4, AU<=4	AI<=3, AU>6	Gaussian	2	213	60	0.28
7	3, 5	AI>3.5, AU<=5	AI<=3.5, AU>5	Gaussian	2	443	198	0.45
8	3, 5	AI>4, AU<=3	AI<=2, AU>6	Gaussian	2	99	27	0.27
9	3, 5	AI>4, AU<=5	AI<=3.5, AU>6	Gaussian	2	291	117	0.40
10	3, 5	AI>4, AU<=5	AI<=3, AU>7	Gaussian	2	72	20	0.28
11	2, 3	AI>3.5, AU<=5	AI<=3.5, AU>5	Gaussian	2	191	70	0.37
12	2, 3	AI>4, AU<=3	AI<=2, AU>6	Gaussian	2	98	32	0.33
13	2, 3	AI>4, AU<=4	AI<=3, AU>6	Gaussian	2	98	32	0.33
14	3, 4	AI>4, AU<=3	AI<=2, AU>6	Gaussian	2	104	28	0.27
15	3,4	AI>4, AU<=4	AI<=3, AU>6	Gaussian	2	223	64	0.29
16	2, 3	AI>4, AU<=5	AI<=3.5, AU>6	Gaussian	2	98	32	0.33
17	3, 4	AI>4, AU<=5	AI<=3.5, AU>6	Gaussian	2	223	64	0.29
18	2, 3	AI>4, AU<=5	AI<=3, AU>7	Gaussian	2	37	7	0.19
19	3, 4	AI>4, AU<=5	AI<=3, AU>7	Gaussian	2	77	22	0.29
20	3, 5	AI>4, AU<=3	AI<=2, AU>6	Minimum value	2	99	27	0.27
21	3, 5	AI>4, AU<=4	AI<=3, AU>6	Minimum value	2	213	59	0.28

Table S3. Variation of analysis strategies for CA1. Table indicates various analysis conditions and the number of (s)CASCs and hits from random analysis (Random1 (R1) in Table S2) where the labels of AU and AI are randomized. Max Missing indicates the maximum number of values missing (after imputation) that are acceptable for a feature. If there are more values missing than Max Missing for either AU or AI, the feature is filtered out. If there are fewer, the feature is retained, but the missing values are not considered (dropped). Imputation (Imp.) 1 and 2 refer to the two possible conditions in which missing values are imputed for the category that possesses more missing values than the other category. These numbers refer to the number of missing values required to trigger imputation. Gaussian imputation replaces missing values with a random value from a gaussian distribution centered around 10000 with a standard deviation of 1000. Minimum value imputation replaces missing values with the minimum value ion intensity detected of any feature. Significant fold-change (sig. FC) refers to the required fold-change in peptide abundance to be considered significant (if the adjusted p-value threshold is also met). The conditions chosen for the main analysis are indicated by the "sCASC" or "CASC" labels on that row (rows are highlighted in light yellow). For DG and CA3, since there are 9 AU instead of 10 AU, the Max Missing for AU is 4 for both the strict and general analysis, and the imputation conditions are AI>4 and AU<=4 missing and AI<=3 and AU>5 missing. This is consistent with the rule that greater than or equal to 66% of values are missing in one condition and that greater than or equal to 55% of values are present in the other condition in order for imputation to occur.

Dataset S1 – protein summary data from cognition experiment, CA1 Region sCASCs

Dataset S2 – protein summary data from cognition experiment, CA1 Region CASCs

Dataset S3 – protein summary data from cognition experiment, DG and CA3 Region

Dataset S4 – protein summary data from refolding experiment
